# Mdm2 enhances ligase activity of parkin and facilitates mitophagy

**DOI:** 10.1038/s41598-020-61796-4

**Published:** 2020-03-19

**Authors:** Seunghyi Kook, Xuanzhi Zhan, Kimberly Thibeault, Mohamed R. Ahmed, Vsevolod V. Gurevich, Eugenia V. Gurevich

**Affiliations:** 1 Department of Pharmacology, Vanderbilt University, Nashville, TN 37232 USA; 2 Department of Pediatrics, Division of Neonatology, Vanderbilt University, Nashville, TN 37232 USA; 3 Department of Chemistry, Tennessee Technological University, Cookeville, TN 38505 USA; 4 Biomaterials and Advanced Drug Delivery Laboratories, Stanford University, Palo Alto, CA 94304 USA

**Keywords:** Enzyme mechanisms, Mitophagy

## Abstract

Loss-of-function mutations in the E3 ubiquitin ligase parkin have been implicated in the death of dopaminergic neurons in the substantia nigra, which is the root cause of dopamine deficit in the striatum in Parkinson's disease. Parkin ubiquitinates proteins on mitochondria that lost membrane potential, promoting the elimination of damaged mitochondria. Neuroprotective activity of parkin has been linked to its critical role in the mitochondria maintenance. Here we report a novel regulatory mechanism: another E3 ubiquitin ligase Mdm2 directly binds parkin and enhances its enzymatic activity *in vitro* and in intact cells. Mdm2 translocates to damaged mitochondria independently of parkin, enhances parkin-dependent ubiquitination of the outer mitochondria membrane protein mitofusin1. Mdm2 facilitates and its knockdown reduces parkin-dependent mitophagy. Thus, ubiquitously expressed Mdm2 might enhance cytoprotective parkin activity. The data suggest that parkin activation by Mdm2 could be targeted to increase its neuroprotective functions, which has implications for anti-parkinsonian therapy.

## Introduction

Parkin was first identified through its association with autosomal recessive juvenile parkinsonism, a familial form of Parkinson's disease (PD) with early onset^1^. Loss-of-function mutations in parkin gene, including exon deletions and rearrangements, as well as nonsense and missense mutations, are commonly associated not only with familial, but also with apparently sporadic early onset PD^2–5^. Functionally, parkin is an E3 ubiquitin ligase^6^. Initially, the studies of parkin function in PD concentrated on the search for parkin substrates and the role of parkin loss in the dysfunction of the ubiquitin-proteasome system (UBS), which appears to play a prominent role in the PD-related neurodegeneration^7^. Further studies implicated parkin in the regulation of the mitochondrial dynamics^8–10^. PINK1, another protein associated with a recessive form of early onset familial PD^11^, acts upstream of parkin, recruiting parkin to damaged mitochondria and stimulating its ligase activity^10,12–15^. Parkin then ubiquitinates proteins of the outer mitochondrial membrane causing their proteosomal degradation and elimination of the damaged mitochondria via mitophagy^16–19^. Mitochondrial dysfunction has been implicated in PD^20–23^, although the role of mitophagy in the quality control of neuronal mitochondria remains controversial^24–26^. Parkin exists in an autoinhibited basal state^27–30^. Its activity is regulated, in addition to the PINK1-dependent phosphorylation^15,31,32^, via a variety of mechanisms including formation of multiprotein signaling complexes^33–35^. Full spectrum of parkin interactions that regulate its activity is not yet understood. However, it is clear that this regulation is complex and often involves scaffolding proteins^36^, which may impact on parkin-dependent control of the mitochondrial dynamics and, consequently, its functions in the normal and diseased brain.

We have recently demonstrated that parkin dose-dependently promotes the association of another E3 ubiquitin ligase, murine double minute oncogene (Mdm2), with arrestins^37^, proteins best known for their role in the homologous desensitization of G protein-coupled receptors (GPCRs)^38^. However, another major function of arrestins is to act as scaffolds of multi-protein complexes regulating the activity of signaling proteins^39–42^. Mdm2, a known oncogene, negatively regulates proapoptotic transcription factor p53 via its ubiquitination and subsequent degradation^43–45^ or via direct interaction with its transactivation domain, inhibiting p53 transcriptional activity^44–47^. Interestingly, parkin was shown to bind Mdm2 substrate p53, and this interaction suppresses parkin-dependent mitophagy^48–50^. Mdm2 was also shown to be imported into mitochondria, where it suppresses respiration and increases invasiveness of cancer cells independently of its role in p53 degradation^51^. Arrestins bound to GPCRs are ubiquitinated by Mdm2, the process that plays a role in the receptor trafficking^52,53^. Furthermore, arrestins recruited to GPCRs bind Mdm2 and reduce Mdm2-mediated degradation of p53, thus promoting p53-dependent apoptosis^54,55^. Interestingly, parkin facilitates the degradation of cytosolic p53, thereby inhibiting apoptosis^56^.

Here we show that parkin directly interacts with Mdm2, and that Mdm2 enhances enzymatic activity of parkin both in the *in vitro* system reconstituted from purified proteins and in intact cells, increasing its self-ubiquitination and ubiquitination of parkin's mitochondrial substrate mitofusin1. Furthermore, we demonstrate that Mdm2 translocates to damaged mitochondria independently of parkin and promotes parkin-dependent mitophagy, revealing a novel mode of parkin activation by direct binding of Mdm2. Thus, Mdm2 stimulates catalytic activity of parkin and promotes its biological functions in intact cells.

## Results

### Mdm2 directly binds parkin and enhances its catalytic activity

**Figure 1:**
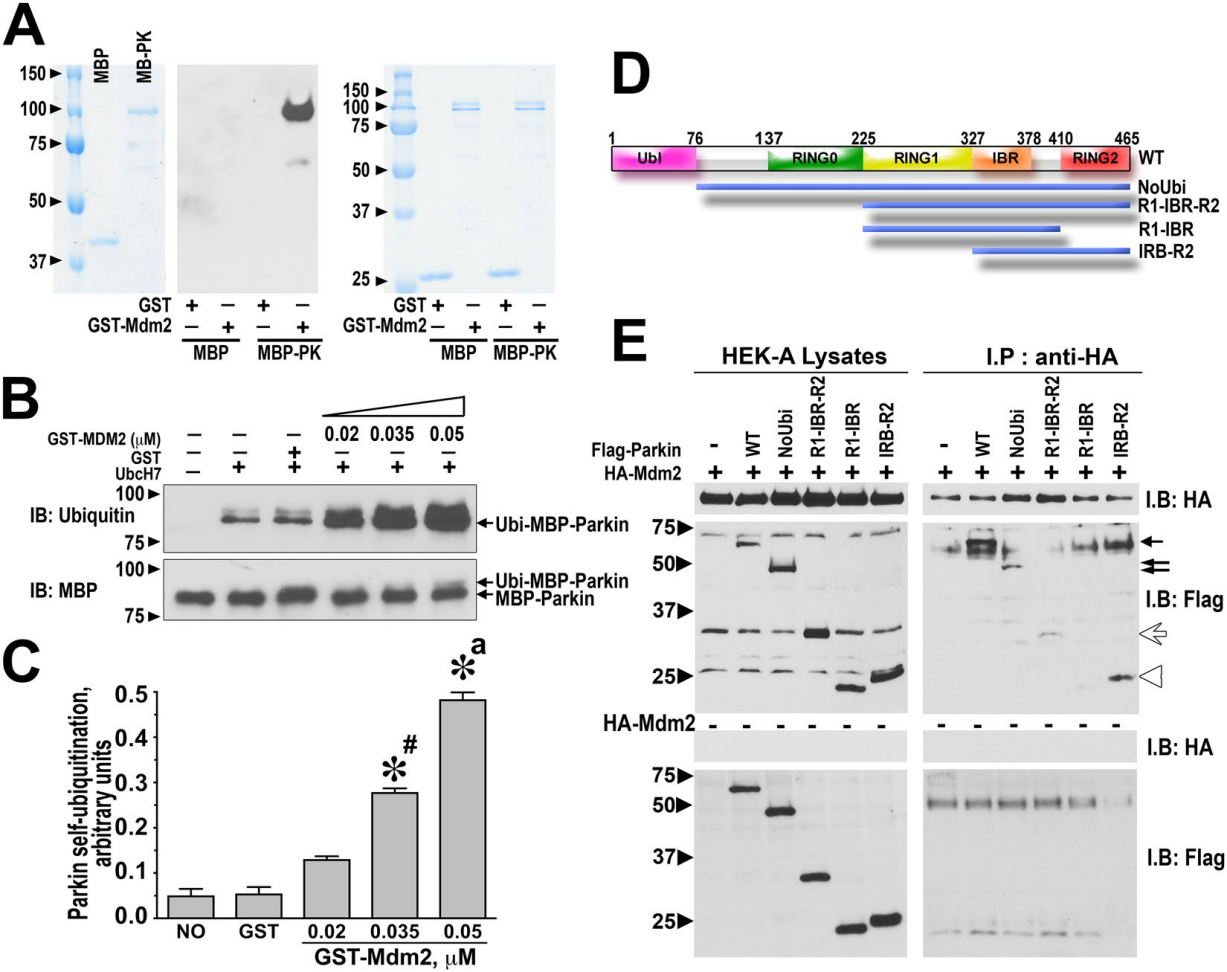
Mdm2 directly binds parkin and enhances its catalytic activity. (**A**) The left panel shows the load of MBP-parkin (MBP-PK) and MBP control by Coomassie staining. The middle panel shows Western blot for MBP-parkin retained by GST-Mdm2, but not by GST control, detected with anti-MBP antibody. The right panel shows equal loading of bait, GST and GST-Mdm2 (Coomassie staining). (**B**) Purified MBP-parkin (0.2 μM) was incubated with ubiquitin, without (negative control) or with the mix of E1 + E2 (UbcH7) ligases and indicated concentrations of GST-Mdm2 (0.02–0.05 μM) in 20 μl for 2 h at 30 °C. The reactions were stopped by 20 μl of SDS buffer. The proteins were resolved by SDS-PAGE and blotted with indicated antibodies. MBP blot shows equal loading of MBP-parkin, ubiquitin blot shows stimulation of parkin activity by GST-Mdm2. As reported previously^58^, only mono-ubiquitination of parkin is detectable *in vitro* at 30 °C, whereas in cells grown at 37 °C multi- and/or poly-ubiquitination yielding a “ladder” is prevalent (Fig. 2). (**C**) Quantification of the data shown in (**B**) from 2 independent experiments. The data were analyzed by one-way ANOVA with Mdm2 as the main factor. *p < 0.001 to NO (buffer in the sample instead of Mdm2) and GST (GST alone 0.05 μM); ^a^p < 0.001 to both 0.05 and 0.035 μM Mdm2; ^#^p < 0.01 to 0.02 μM Mdm2 by Bonferroni host hoc test with correction for multiple comparisons. (**D**) The domains of parkin and constructs with domain deletions used in the immunoprecipitation experiments. (**E**) Immunoprecipitation of isolated parkin domains by full-length Mdm2. Left middle panel shows the expression of HA-Mdm2 and Flag-parkin (lane 1 – negative control without parkin) in HEK293 cell lysates. Right middle panel: HA-Mdm2 was IPed with anti-HA antibody and co-IPed Flag-parkin constructs were detected by Western blot with anti-Flag antibody. All constructs containing R2 (full-length WT, arrow; parkin lacking Ubl, double arrow; R1-IBR-R2, white arrow; IBR-R2, white arrowhead) bound Mdm2, whereas R1-IBR did not (detected in lysate, but not in IP sample). Lower panels – no bate (no HA-Mdm2) negative controls. Note that the two bands visible in the negative control are non-specific IgG.

We previously demonstrated that parkin binds arrestins and promotes the recruitment of Mdm2 into the complex^37^. Therefore, we tested whether parkin directly binds Mdm2 without intermediaries. Direct binding can only be proved by the demonstration that two purified proteins interact. First, we used *in vitro* pull-down assay with purified recombinant parkin tagged with maltose binding protein (MBP) and GST-Mdm2 (Fig. 1A). Equal amounts of GST-Mdm2 or GST (control) were immobilized on glutathione column, and the ability of these proteins to retain MBP-parkin was determined (MBP was used as a negative control). We found that GST-Mdm2, but not GST, retained MBP-parkin, whereas neither protein interacted with MBP (Fig. 1A). Thus, parkin can bind Mdm2 directly in the absence of arrestins or other proteins.

Parkin self-ubiquitinates, and its self-ubiquitination has been used as readout for its ligase activity^57,58^. Therefore, we tested the effect of Mdm2 binding on parkin self-ubiquitination. To ascertain that the effect is direct, rather than mediated by other proteins, we performed *in vitro* experiments with purified MBP-parkin and GST-Mdm2, where GST served as a control. As can be expected for an E3 ubiquitin ligase, parkin activity depends on the presence of a mix of E1/E2 ubiquitin ligases (Fig. 1B). GST-Mdm2 dose-dependently increased parkin self-ubiquitination, whereas GST has no effect (Fig. 1B). As was previously shown *in vitro*, in these experimental conditions parkin monoubiquitinates^58^. In these experiments, we used the E2 ligase UbcH7 that has broad specificity for HECT type E3 ligases but does not interact with RING type E3 ligases, including Mdm2^59,60^. Since parkin possesses mixed qualities of the RING and HECT ligases^30,61^, UbcH7 effectively interacts with parkin^62^. Thus, we concluded that parkin self-ubiquitinated at a higher level in the presence of Mdm2 rather than was ubiquitinated by Mdm2, i.e., the binding of Mdm2 enhances enzymatic activity of parkin.

Parkin is a multi-domain protein^27,28,30^. In order to determine which parkin elements mediate its interactions with Mdm2, we generated a series of Flag-tagged parkin constructs ranging from full-length to separated rings 1 and 2 (R1 and R2, respectively) with in-between-rings (IRB) linker (Fig. 1C). These constructs were co-expressed with HA-tagged Mdm2, whereupon Mdm2 was immunoprecipitated with anti-HA antibody, and co-immunoprecipitated forms of parkin were visualized by Western blot with anti-Flag antibody. Full-length parkin and IBR-R2 demonstrated the most avid binding, followed by parkin lacking ubiquitin-like domain and R1-IBR-R2 construct (Fig. 1C). Since R1-IBR element did not interact with Mdm2, these data suggest that R2 is the main Mdm2-binding element of parkin. Interestingly, ubiquitin ligases containing ring domains often dimerize via these elements. In particular, ring domain of Mdm2 was shown to mediate its self-dimerization and interaction with MdmX^63,64^. Thus, it appears likely that ring-ring interaction mediates the binding of Mdm2 and parkin.

### Mdm2 increases parkin activity in intact cells

**Figure 2:**
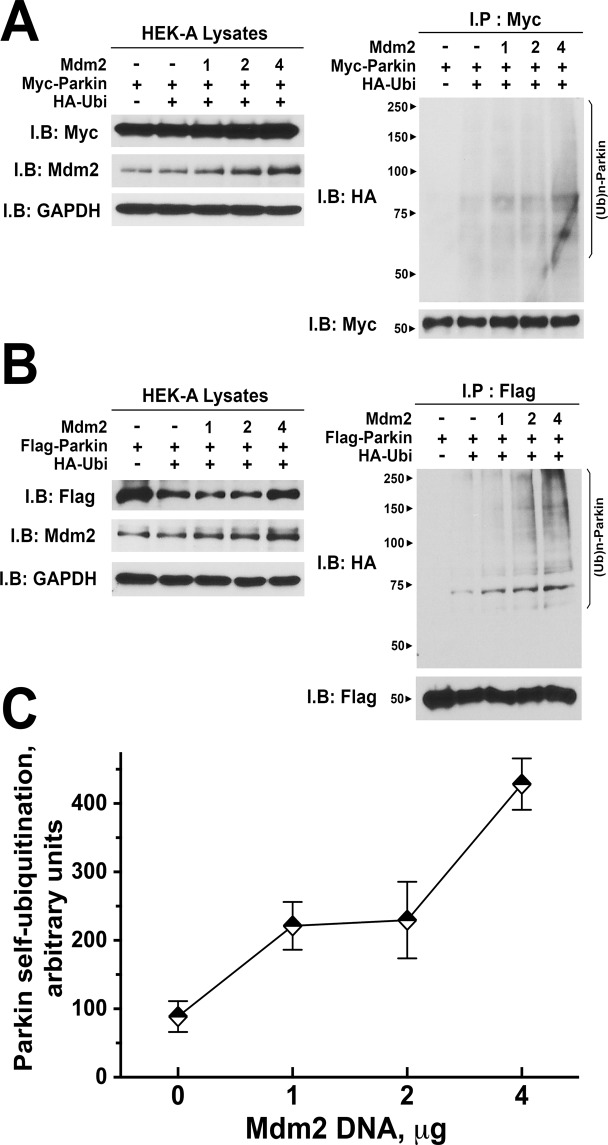
Mdm2 dose-dependently increases parkin self-ubiquitination in intact cells. HEK293A cells were transfected with HA-ubiquitin, myc- (**A**) or FLAG-parkin (**B**), and varying amounts of untagged Mdm2. Parkin was immunoprecipitated with anti-myc or anti-FLAG antibody, and its ubiquitination was determined by Western blot with anti-HA antibody. Note that Mdm2 progressively increases the ubiquitination of WT parkin. (**C**) Quantification of the level of parkin self-ubiquitination in the presence of different concentrations of Mdm2 from four independent experiments. Data are presented as means + S.E.M. ANCOVA analysis with Mdm2 concentration as a co-variate yielded significant effect of Mdm2 (p < 0.0001).

Next, we tested whether Mdm2 increases parkin activity in the context of an intact cell. To this end, we co-expressed Myc-tagged parkin with HA-tagged ubiquitin and increasing amounts of untagged Mdm2 in HEK293 cells (Fig. 2A). Samples of immunoprecipitated parkin were blotted for HA to determine the extent of its ubiquitination. We found that Mdm2 dose-dependently promoted incorporation of ubiquitin into parkin (Fig. 2A). Mdm2 similarly increased ubiquitination of Flag-tagged parkin (Fig. 2B), demonstrating that the effect does not depend on protein tags. Statistical analysis revealed clear correlation between Mdm2 expression and ubiquitin incorporation into parkin (Fig. 2C). Interestingly, in-cell parkin ubiquitination generated a characteristic “ladder”, indicating the presence of multi- and/or poly-ubiquitinated parkin (Fig. 2A,B), whereas parkin self-ubiquitination *in vitro* was largely limited to mono-ubiquitination, as described previously^58^ (Fig. 1B).

**Figure 3:**
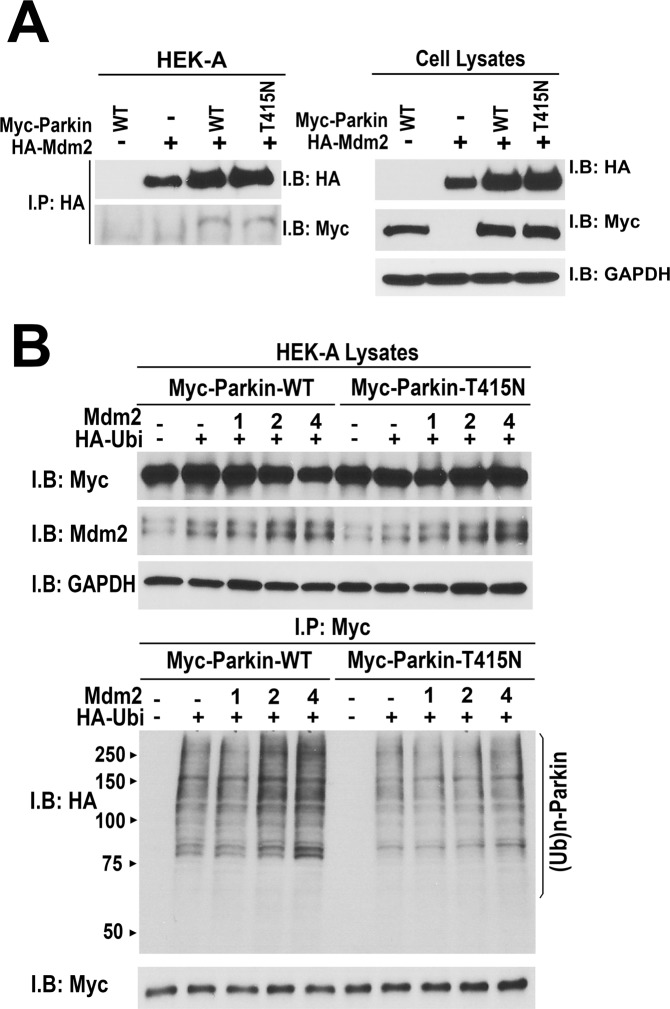
Mdm2-dependent increase of parkin ubiquitination requires catalytically active parkin. (**A**) HEK293 cells were transfected with myc-parkin or catalytically inactive myc-parkin-T415N mutant and HA-Mdm2. Mdm2 was immunoprecipitated with anti-HA antibody, and the samples were blotted for HA and myc. The results (left panel) show that both WT and mutant parkin interact with Mdm2. Right panel shows the expression of myc-parkin constructs and HA-Mdm2 in cell lysates. (**B**) HEK293A cells were transfected with HA-ubiquitin, myc-parkin or catalytically inactive myc-parkin-T415N mutant, and varying amounts of untagged Mdm2. Parkin was immunoprecipitated with anti-myc antibody, and its ubiquitination was determined by Western blot with anti-HA antibody. Upper panels show the expression of myc-parkin and untagged Mdm2 in cell lysates. Lower panel shows ubiquitination of parkin detected with anti-HA antibody in immunoprecipitated parkin (upper blot) and the equal load of immunoprecipitated myc-parkin detected with anti-myc antibody. Note that Mdm2 progressively increases the ubiquitination of WT parkin, but has no effect on catalytically inactive T415N mutant, which binds Mdm2 as well as WT parkin, demonstrating that Mdm2 enhances parkin self-ubiquitination.

Since Mdm2 is an E3 ubiquitin ligase potentially capable of ubiquitinating parkin in cells, where the full complement of E2 ubiquitin ligases is available, increased incorporation of ubiquitin into parkin in the presence of Mdm2 could reflect Mdm2-dependent ubiquitination of parkin, rather than increased parkin self-ubiquitination. To test whether this is the case, we used ligase-dead mutant parkin-T415N that does not self-ubiquitinate but retains normal ability to bind Mdm2 (Fig. 3A). We found that the incorporation of ubiquitin into immunoprecipitated parkin-T415N from HEK293 cells co-expressing increasing amounts of Mdm2 was low, regardless of the Mdm2 concentration (Fig. 3B), even though the expression of wild type (WT) parkin and its T415N mutant was balanced, and HA-Mdm2 was expressed at the same levels (Fig. 3B). Thus, in this paradigm the incorporation of ubiquitin into parkin requires its own enzymatic activity and cannot be attributed to its ubiquitination by Mdm2 and/or other ubiquitin ligases present in cells.

### Mdm2 enhances parkin activity towards endogenous substrate mitofusin1

**Figure 4:**
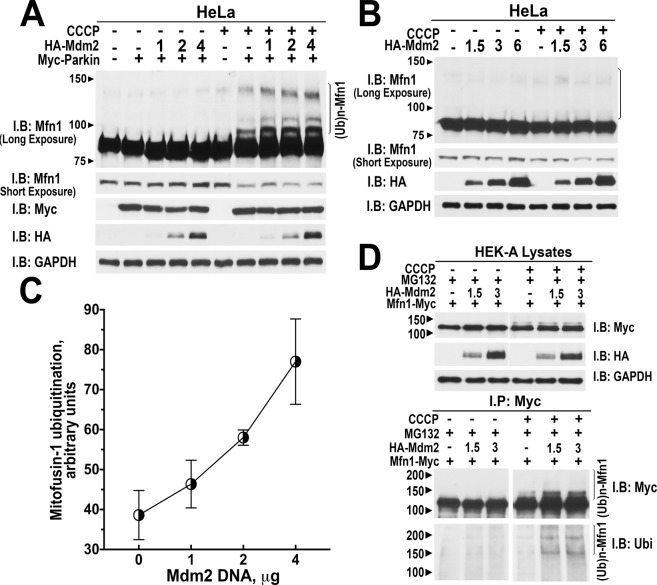
Mdm2 promotes ubiquitination of mitofusin1 by parkin in different cell types. (**A**) HeLa cells that do not express endogenous parkin were transfected with myc-parkin and increasing amounts of HA-Mdm2 and treated with CCCP (10 μM; 3 h). Cell lysates were analyzed by Western blot with anti-mitofusin1 antibody to detect ubiquitination of endogenous mitofusin1. Ubiquitinated mitofusin1 (Mfn1) species are indicated with the bracket on the right. Middle and lower blots show the expression of myc-parkin (anti-myc antibody) and increasing levels of HA-Mdm2 (anti-HA antibody). Note that the presence of parkin induced mitofusin1 ubiquitination, and this effect was further enhanced by Mdm2. (**B**) HeLa cells transfected with increasing amounts of HA-Mdm2 were treated with CCCP (10 μM; 3 h). Cell lysates were analyzed by Western blot with anti-mitofusin1 antibody to detect ubiquitination of endogenous mitofusin1 (Mfn1). The area where ubiquitinated mitofusin1 species were detected in panel A is indicated with the bracket on the right. Middle and lower blots show the increasing levels of HA-Mdm2 (anti-HA antibody). Note that in the absence of parkin mitofusin1 ubiquitination is not affected by Mdm2. (**C**) Quantification of mitofusin1 ubiquitination in the presence of different concentrations of Mdm2 from three independent experiments. Data are presented as means + S.E.M. ANCOVA analysis with Mdm2 concentration as a co-variate yielded significant effect of Mdm2 (p = 0.009). (**D**) HEK293A cells transfected with myc-mitofusin1 and increasing amounts of HA-Mdm2 were treated with CCCP (10 μM; 1 h). Proteasome inhibitor MG132 (10 μM) was added 30 min before CCCP. Mitofusin1 was immunoprecipitated with anti-myc antibody. Cell lysates were analyzed by Western blot with anti-myc and anti-HA antibodies to detect expression of myc-mitofusin1 and HA-Mdm2. Immunoprecipitates (lower panel) were analyzed by Western blot with anti-myc and anti-ubiquitin antibodies to detect ubiquitination of mitofusin1 by endogenous parkin. Ubiquitinated mitofusin1 species are indicated with the bracket on the right. Note that mitofusin1 ubiquitination by endogenous parkin was dose-dependently enhanced by Mdm2. GAPDH was used as loading control.

Parkin self-ubiquitination, while a convenient readout, may not fully reflect biologically relevant ubiquitin ligase activity of parkin. In order to determine the functional significance of the Mdm2 effect on parkin, we tested whether Mdm2 facilitates parkin-dependent ubiquitination of other intracellular substrates. Parkin translocates to the mitochondria with lost membrane potential in PINK1-dependent manner and ubiquitinates proteins of the outer mitochondrial membrane, including mitofusins 1 and 2, to induce autophagy of damaged mitochondria^17,18^. Therefore, we tested whether Mdm2 promotes the ubiquitination of mitofusin1, a well-established parkin substrate, upon mitochondria depolarization. In HeLa cells lacking endogenous parkin, we co-expressed myc-parkin and increasing amounts of HA-tagged Mdm2, treated cells with carbonylcyanide *m*-chlorophenylhydrazone (CCCP) to depolarize mitochondria or vehicle as a control, and blotted for endogenous mitofusin1 with anti-mitofusin1 antibody (Fig. 4A). We found that the level of mitofusin1 ubiquitination increased in the presence of Mdm2 in cells treated with CCCP, but not in untreated cells, where no ubiquitinated mitofusin1 was detected (Fig. 4A). Importantly, the expression of Mdm2 alone without parkin did not induce mitofusin1 ubiquitination regardless of CCCP treatment (Fig. 4B), demonstrating that parkin, but not Mdm2 and/or other ubiquitin ligases present in HeLa cells, is the ubiquitin ligase modifying mitofusin1. In CCCP-treated cells mitofusin1 ubiquitination progressively increased with the level of expressed Mdm2 (Fig. 4C).

To exclude artifacts due to over-expression, next we tested the effect of Mdm2 on ubiquitination of mitofusin1 by endogenous parkin in HEK293 cells. To this end, myc-tagged mitofusin1 was co-expressed with increasing amounts of HA-Mdm2. Cells were treated with CCCP or vehicle, in the presence of MG132, an inhibitor of proteasomal degradation, then mitofusin1 was immunoprecipitated with anti-myc antibody, and samples were immunoblotted for ubiquitin and mitofusin1 with respective antibodies. We found that ubiquitination of mitofusin1 was detected only in cells treated with CCCP; it was elevated by co-expressed Mdm2 in a dose-dependent manner (Fig. 4D). As expression of Mdm2 alone did not enhance mitofusin 1 ubiquitination (Fig. 4B), we concluded that Mdm2-dependent increase in parkin activity leads to biologically relevant tagging of damaged mitochondria via mitofusin ubiquitination by parkin in different cell types.

**Figure 5:**
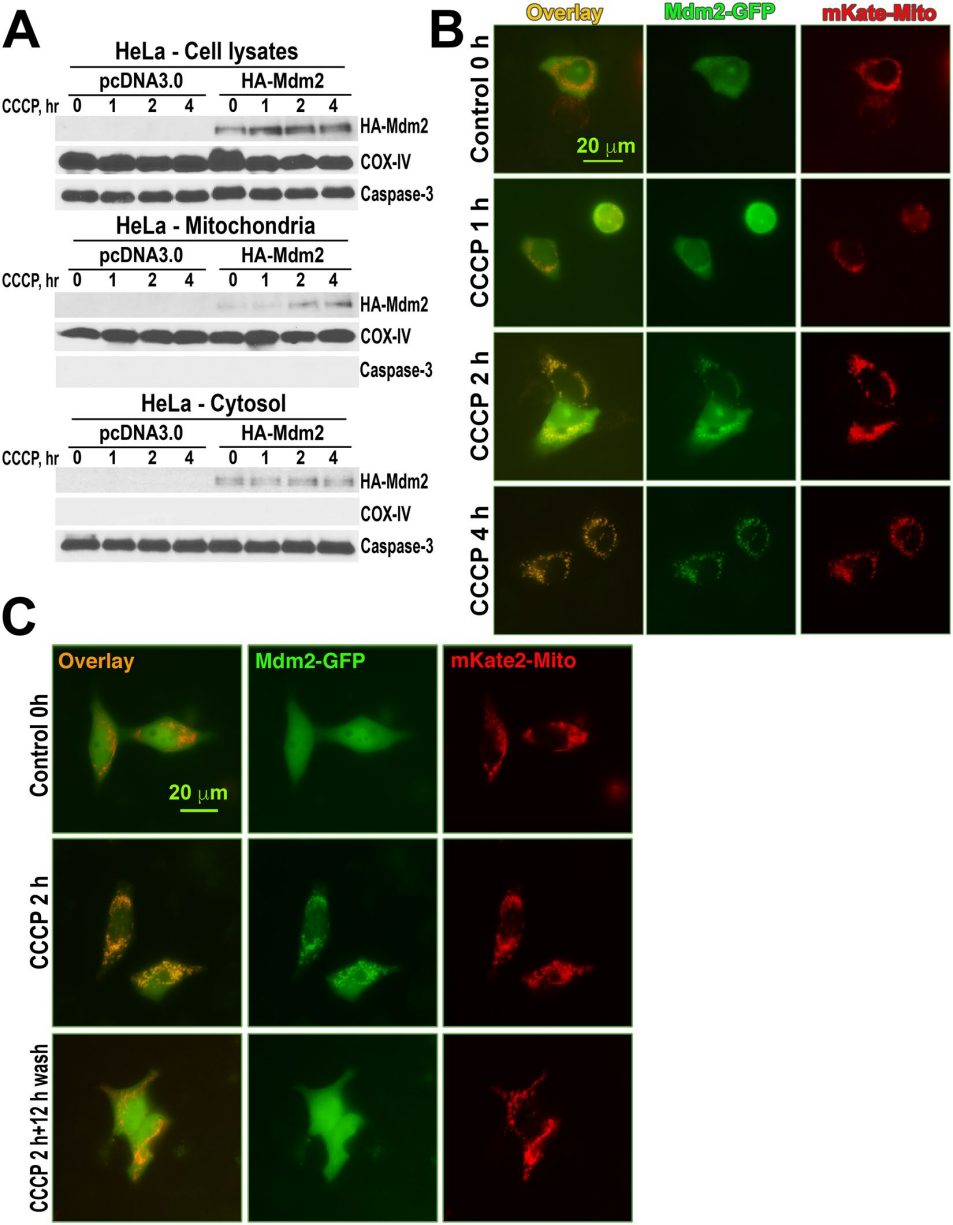
CCCP promotes reversible Mdm2 recruitment to mitochondria. (**A**) HeLa cells transfected with the empty vector or HA-Mdm2 were treated with CCCP (10 μM) for indicated times. Cell lysates were collected, and cytosol and mitochondrial fractions were separated, as described in Methods. The lysates and fractions were blotted for HA to detect HA-Mdm2. COX-IV and caspase-3 served as markers for the mitochondrial and cytosolic fractions, respectively. (**B**) HeLa cells transfected with Mdm2-GFP and mKate-mito were treated with CCCP (10 μM) for indicated times. (**C**) HeLa cells expressing mKate-mito and Mdm-GFP were treated with 10 μM CCCP for 2 h, whereupon CCCP-containing medium was removed and replaced with CCCP-free medium, in which the cells were incubated ~12 h. Cells were imaged live with Nikon microscope LC2000 and 40X oil objective. In panels (**B**,**C**) representative cells co-expressing both proteins (from 2–3 independent experiments performed) are shown.

It is known that parkin is recruited to damaged mitochondria^8,12^. However, the recruitment of Mdm2 to this compartment in response to the loss of mitochondria membrane potential was never reported. Yet to facilitate the activity of parkin towards mitofusin1, some of Mdm2 must localize near parkin, i.e., at the mitochiondria. To determine whether Mdm2 can move to damaged mitochondria under its own power, independently of parkin, we used HeLa cells lacking endogenous parkin, that were treated with CCCP to depolarize mitochondria for varying periods of time (Fig. 5A). Cells expressing HA-Mdm2 (or empty vector as a control) were fractionated, and the presence of HA-Mdm2 in total cell lysates, cytosol, and mitochondria was determined by Western blot (Fig. 5A). Caspase-3 and COX-IV were used as markers of cytosol and mitochondria, respectively. We found that while overall levels of Mdm2 did not appreciably change with time in this experiment, its presence in mitochondria fraction gradually increased, reaching significant levels after two and especially four hours of CCCP treatment (Fig. 5A). We confirmed these results by observations of recruitment of GFP-tagged Mdm2 to mKate-Mito-labeled mitochondria in living cells (Fig. 5B). Thus, Mdm2 is recruited to mitochondria that have lost their membrane potential, and this relocation does not require the presence of parkin. To test whether Mdm2 recruitment to mitochondria is reversible, we treated HeLa cells with CCCP for 2 h to achieve detectable recruitment, and then removed CCCP and incubated cells in normal media. We found that the presence of Mdm2-GFP at mitochondria (labeled by co-expressed mKate-mito) was diminished as early as 2 h after the beginning of the washout. However, at that time point most mitochondria still displayed abnormal morphology. The morphology was not fully restored until after a 12 h washout, at which point Mdm2 essentially returned to its initial cytosolic/nuclear localization (Fig. 5C).

### Mdm2 enhances the removal of damaged mitochondria only in cells expressing parkin

**Figure 6:**
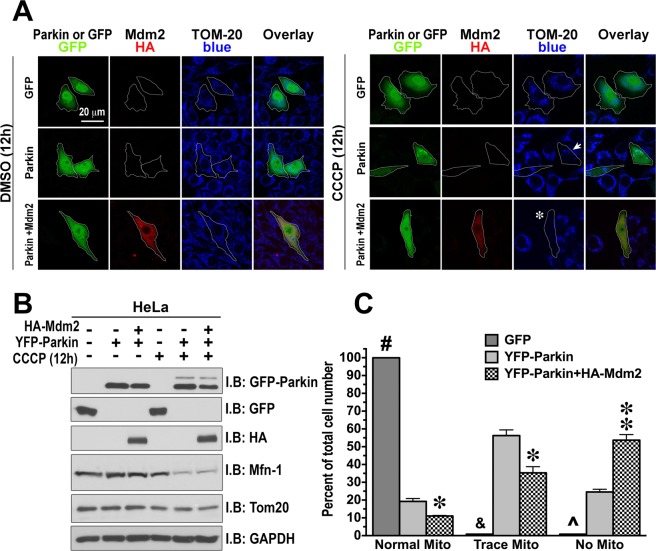
Mdm2 promotes mitophagy of damaged mitochondria. HeLa cells were transfected with GFP, YFP-parkin, or YFP-parkin and HA-Mdm2. Cells were treated for 12 h with either DMSO or CCCP (10 μM) before fixation, and stained with anti-GFP (green), anti-HA (red) and Tom20 (blue) antibodies to label cells expressing parkin, Mdm2, and to visualize the mitochondria. The mitochondrial morphology was evaluated in cells expressing different constructs, and cells were classified as having normal, trace, or no mitochondria. Asterisk indicates a cell with complete and a small arrow – with partial loss of the mitochondria. Note that no change in the mitochondria morphology occurs following the DMSO treatment or without parkin expression. Also note that co-expression of Mdm2 facilitates the loss of the mitochondria following the CCCP treatment producing more cells with no mitochondria. (**A**) Representative images of cells are shown. (**B**) Western blots showing the expression levels of different proteins in control (DMSO-treated) and CCCP-treated HeLa cells used in the imaging experiments shown in **A**. (**C**) Quantification of the mitophagy data. 120–150 cells for each condition from 3 independent transfection experiments were scored. The data were statistically analyzed by one-way ANOVA with Protein as main factor followed by Bonferroni/Dunn post hoc comparison of means with correction for multiple comparisons. ^#^p < 0.001 to parkin and parkin + Mdm2; ^p < 0.01 to parkin and p < 0.001 to parkin + Mdm2; ^&^p < 0.01 to parkin and parkin + Mdm2; *p < 0.05, **p < 0.01 between parkin and parkin + Mdm2.

Ubiquitination of mitofusins by parkin with their subsequent degradation is required for mitophagy^9,18,65^. Therefore, we tested whether enhanced parkin-dependent ubiquitination of mitofusin1 promotes the removal of damaged mitochondria from the cell. To ascertain parkin role in mitophagy, we used HeLa cells lacking endogenous parkin, where we expressed either YFP-parkin or GFP as a control. The cells were co-transfected with HA-Mdm2 (or empty vector as a control). The cells were then treated for 12 h with CCCP (dissolved in DMSO; final concentration 10 μM) to depolarize mitochondria, or the same amount of DMSO as a control, whereupon the cells were fixed. YFP-parkin and GFP were visualized using their own fluorescence (green), HA-Mdm2 was visualized with anti-HA antibody followed by AlexaFluor 594 chicken anti-mouse secondary antibody (red), and mitochondria were visualized with anti-Tom-20 antibody with Dylight 405-conjuated donkey anti-rabbit secondary antibody (blue) (Fig. 6A). The expression of YFP-parkin and GFP was balanced, and HA-Mdm2 was also expressed at the same level in control cells and those treated with CCCP (Fig. 6B). Mitochondria loss was detected only in CCCP-treated cells expressing parkin, as judged by TOM-20 staining of fixed cells (Fig. 6A) and by the amount of mitochondrial proteins mitofusin1 and TOM-20 detected by Western blot (Fig. 6B). After 12 h of treatment with CCCP, YFP-Parkin eliminated all detectable mitochondria from ~25% of cells, as compared to ~54% of cells co-expressing HA-Mdm2 and YFP-Parkin that displayed complete mitochondrial loss (Fig. 6C). Thus, in cells co-expressing YFP-Parkin and HA-Mdm2 mitophagy is dramatically enhanced. Mdm2 does not affect mitochondria survival by itself but promotes parkin-mediated degradation of damaged mitochondria (Fig. 6). These data suggest that Mdm2-dependent increase in parkin enzymatic activity in intact cells translates into facilitated removal of mitochondria lacking membrane potential.

### Mdm2 knockdown impedes the parkin-dependent removal of damaged mitochondria

**Figure 7:**
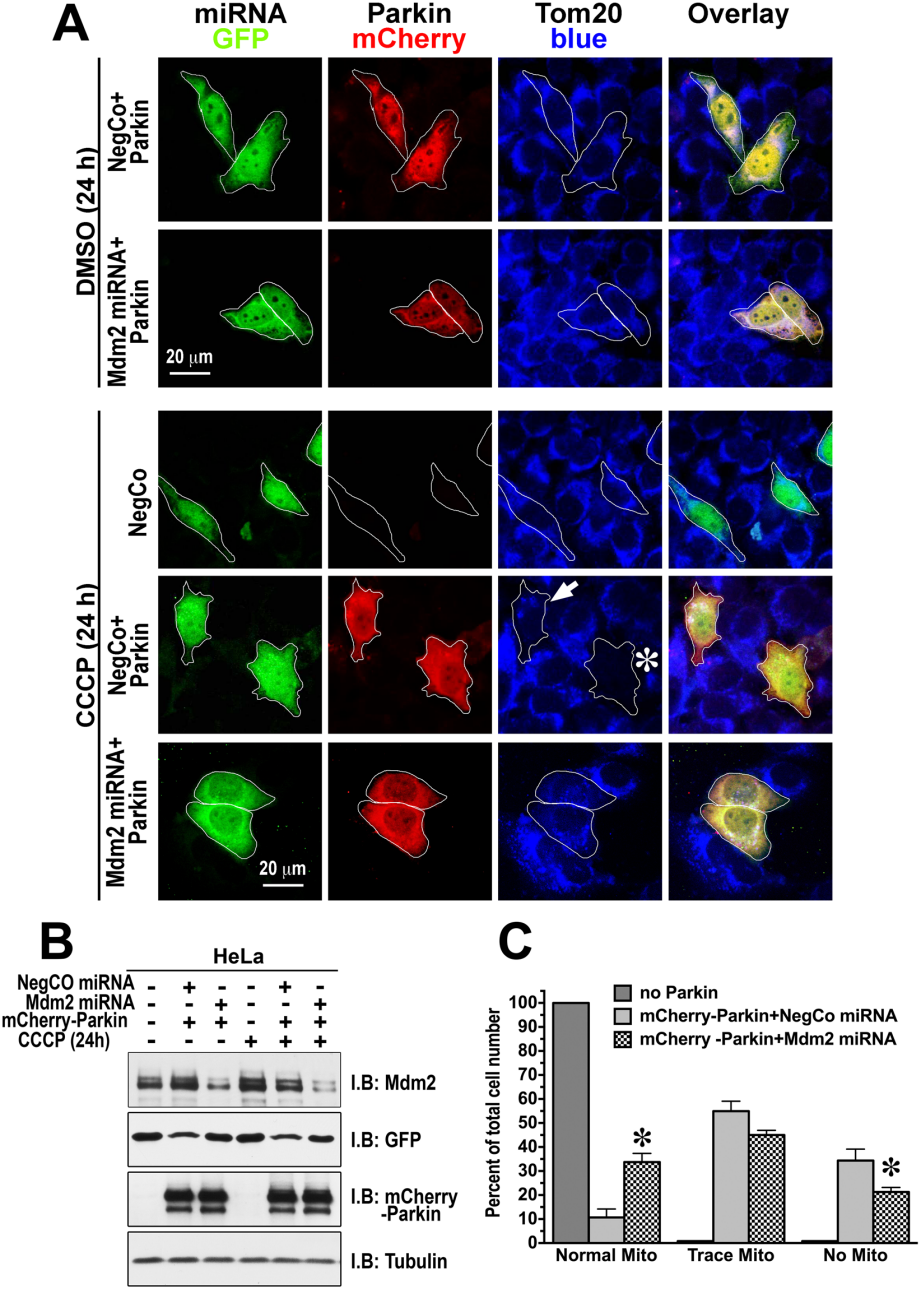
Mdm2 knockdown impedes mitophagy of damaged mitochondria. HeLa cells were transfected with negative control miRNA or Mdm2 miRNA (both co-cistronically expressing GFP) with or without mCherry-parkin. Cells were treated for 24 h with either DMSO or CCCP (10 μM) before fixation, and stained with GFP (green), mCherry (red), and Tom20 (blue) antibodies to label cells expressing parkin, negative control or Mdm2 siRNA, and to visualize the mitochondria. The mitochondrial morphology was evaluated in cells expressing different constructs, and cells were classified as having normal, trace, or no mitochondria. The outlines in the panels for parkin and mitochondria show the position of cells expressing miRNA. Asterisk indicates a cell with complete and a small arrow – with partial loss of the mitochondria. Note that cells expressing NegCo + PK treated with CCCP show little or no mitochondria, whereas cells expressing Mdm2 miRNA largely retain their mitochondria. (**A**) Representative images of cells are shown. (**B**) Western blots showing the expression levels of different proteins in control (DMSO-treated) and CCCP-treated HeLa cells used in the imaging experiments shown in **A**. (**C**) Quantification of the mitophagy data. 150–200 cells for each condition from 3 independent transfection experiments were scored. The data were statistically analyzed by one-way ANOVA with Protein as main factor followed by Bonferroni/Dunn post hoc comparison of means with correction for multiple comparisons. *p < 0.05 between negative control miRNA + parkin and Mdm2 miRNA + parkin.

To further explore the role of Mdm2 in parkin-dependent mitophagy, we used HeLa cells lacking endogenous parkin, where we expressed or omitted mCherry-parkin. The cells were co-transfected with negative control miRNA or Mdm2 miRNA. We first examined mCherry-parkin clustering at the mitochondria upon CCCP treatment in cells expressing negative control miRNA or Mdm2 miRNA (Fig. 7) and found that Mdm2 knockdown tends to slow down parkin recruitment. Next, we treated the cells expressing negative control miRNA or Mdm2 miRNA with or without mCherry-parkin for 24 h with CCCP (dissolved in DMSO; final concentration 10 μM) to depolarize mitochondria, or the same amount of DMSO as a control, whereupon the cells were fixed. mCherry-parkin and GFP (co-cistronically expressed with miRNAs) were visualized with specific primary antibodies followed by Alexa594 (red)- or Alexa488 (green)-conjugated secondary antibodies, respectively, and mitochondria were visualized with anti-Tom-20 antibody with Dylight 405-conjuated donkey anti-rabbit secondary antibody (blue) (Fig. 7A). The expression of negative control and Mdm2 miRNA is shown by the presence of co-cistronically expressed GFP, and the level of mCherry-parkin was also balanced across experimental conditions (Fig. 7B). Mitochondria loss was detected only in CCCP-treated cells expressing parkin, as judged by TOM-20 staining of fixed cells (Fig. 7A). After 24 h of treatment with CCCP, only ~21% of cells co-expressing Mdm2 miRNA and mCherry-Parkin displayed complete mitochondrial loss, whereas ~34% of cells co-expressing negative control miRNA and mCherry parkin did so (Fig. 7C). The Mdm2 knockdown also resulted in a significant increase in the percentage of cells with normal mitochondria: from ~11% to ~34%. These data, together with the results on Mdm2 overexpression, suggest that Mdm2-dependent increase in parkin enzymatic activity is an important factor regulating the mitochondrial dynamics and mitophagy.

## Discussion

The key finding of this study is that E3 ubiquitin ligase Mdm2 plays a direct role in mitophagy by regulating the activity of another E3 ubiquitin ligase, parkin, discovered via its genetic link to PD, as the name implies. Although the role of Mdm2 in mitochondria function was proposed earlier^66^, in that study Mdm2 appeared to act via ubiquitination of its well-established substrate, p53. Another reported mitochondria-related function of Mdm2 was not connected with p53 ubiquitination, involving instead its import into these organelles, where Mdm2 appears to repress the transcription of NADH-dehydrogenase 6, inhibiting respiratory complex I activity^51^. Here we report that Mdm2 directly binds parkin increasing its enzymatic activity. This effect was documented with purified proteins, where indirect regulation or association via intermediaries can be excluded. We also found that Mdm2 increases parkin activity in cells, thereby facilitating mitophagy independently of its action on p53 (Figs. 6 and 7). The finding that Mdm2 does not increase mitofusin1 ubiquitination in the absence of parkin (Fig. 4B) suggests that mitofusin1 is ubiquitinated by parkin, not by Mdm2 and/or other ubiquitin ligases present in HeLa cells. We also found that Mdm2 is recruited to damaged mitochondria and its recruitment is independent of parkin, for it was observed in HeLa cells lacking parkin (Fig. 5). Washout of CCCP, which depolarizes mitochondria, reverses Mdm2 recruitment (Fig. 5C). This Mdm2 recruitment is clearly different from reported Mdm2 import into the mitochondria^51^, as recruited parkin is known to localize to the outer mitochondrial membrane, where it ubiquitinates its substrates, so that to enhance parkin activity, Mdm2 must be also localized to the outer shell of mitochondria. Importantly, parkin appears to be recruited to the mitochondria independently of Mdm2, since the Mdm2 recruitment to the mitochondria seems to lag behind that of parkin. These findings suggest that ubiquitously expressed Mdm2 might play a role in parkin-dependent mitochondria quality control. It is important to note that while Mdm2 significantly enhances parkin activity *in vitro* (Fig. 1) and in cells (Figs. 2 and 4), it is not required for parkin activity. Thus, Mdm2-dependent parkin activation is a regulatory mechanism, not an on-off switch.

Parkin is recruited to damaged mitochondria^12,13^, where it ubiquitinates outer membrane proteins promoting mitophagy. Ubiquitination of mitofusins by parkin with their subsequent degradation was reported to stimulate mitophagy, which is believed to be a cytoprotective process that eliminates damaged mitochondria^9,18,65^. Indeed, CCCP treatment of HeLa cells induced mitophagy strictly in parkin-dependent manner. Co-expression of Mdm2 with parkin doubled the fraction of cells that completely eliminated their mitochondria upon CCCP treatment. Thus, direct binding of Mdm2 to parkin enhances parkin ligase activity in the biologically relevant manner, resulting in enhanced parkin-mediated ubiquitination of the outer membrane proteins and more pronounced mitophagy.

We found that Mdm2 enhanced in a dose-dependent manner the ubiquitination of mitofusin1, one of the best-established parkin substrates^17^, by exogenously expressed or endogenous parkin in cells treated with mitochondria-depolarizing agent CCCP. Importantly, in the absence of parkin Mdm2 itself does not increase mitofusin1 ubiquitination (Fig. 4B), indicating that neither Mdm2, nor other ubiquitin ligases present in HeLa cells can perform this function. Mdm2 enhanced catalytic activity of parkin towards mitofusin1 in different cell types, suggesting general biological importance of this phenomenon. The dose-dependence of the effect suggests that the interaction of these two ubiquitin ligases is regulated by their local concentrations.

One caveat of the study is that the readout was the disappearance of the mitochondria following treatment with the uncoupling agent CCCP. The steady-state level of the mitochondria could be affected, in addition to elimination by mitophagy, by *de novo* biogenesis. Thus, our findings that Mdm2 promotes the loss of mitochondria upon the CCCP treatment could be due to enhanced mitophagy, suppressed biogenesis, or both. We have not analyzed the rate mitochondria biogenesis in HeLa cells under our experimental conditions due to the lack of tools compatible with our experimental design. Although there remains a possibility that suppression of the mitochondria biogenesis contributed to the observed results, it does not appear likely. First, it is important to note that the changes in the mitochondria were strictly dependent on the presence of parkin and were absent in control parkin-deficient HeLa cells. Second, the only role of parkin in mitochondria biogenesis demonstrated to date is to *promote* it via parkin's ligase actions^67,68^.

While we cannot formally exclude the possibility that Mdm2 promoted parkin-dependent mitophagy and at the same time suppressed mitochondria biogenesis in parkin-dependent manner, this scenario does not appear likely. Parkin had been implicated in elimination of damaged mitochondria by mitophagy but has never been shown to *inhibit* the mitochondria biogenesis. Furthermore, the role of parkin in promoting the mitochondria biogenesis has been demonstrated in neuronal cells and in the brain of living animals^67,68^, and the conditional knockout of parkin results in the loss of mitochondria and resulting neuronal degeneration^68^. HeLa cells used in this study have been derived from cervical cancer cells and lack parkin, as do many cancer cells, in which parkin acts as a tumor suppressor^69,70^. We show that Mdm2 enhances parkin's ligase activity and parkin-dependent ubiquitination of mitofusin-1, one of the key regulators of mitophagy^71^.Thus, the most straightforward interpretation of the evidence is that Mdm2 facilitates parkin-dependent mitophagy.

Mitochondria dysfunction was identified as a probable cause of neuronal death in PD^22,72^. Parkin was implicated in mitochondria maintenance, particularly in elimination of damaged mitochondria, which is triggered by the ubiquitination of PINK1-phosphorylated mitofusins by parkin^17,73^. Parkin directly binds tumor suppressor p53, which is the best-characterized substrate of Mdm2. It appears that p53 binding impedes parkin recruitment to damaged mitochondria, thereby inhibiting its function in mitochondria maintenance^48–50^. Parkin appears to play a complex role in apoptotic cell death. On the one hand, loss of parkin inhibits p53 degradation^74^, thereby promoting p53-dependent apoptosis. On the other hand, parkin promotes ubiquitination and degradation of cytosolic cytochrome c, thereby increasing cell survival^56^.

Mdm2 was so far never directly associated with any aspect of the parkin function. Like parkin, Mdm2 is an E3 ubiquitin ligase. It is best known as a regulator of pro-apoptotic transcription factor p53: it ubiquitinates p53 and promotes its degradation^43–45^ or inhibits its activity via direct binding to its transactivation domain^46,47,75^. Existing evidence suggests that ubiquitination-dependent mechanism of p53 regulation by Mdm2 is the most important. The role of p53 in the cytoprotective function of Mdm2 was demonstrated by the finding that Mdm2 knockout in mice is embryonic lethal, whereas simultaneous knockout of Mdm2 and p53 yields viable animals^76^. Mdm2 binds arrestins^52^, which are ubiquitous regulators of cell signaling first discovered for their role in homologous desensitization of G protein-coupled receptors^39,41^. Mdm2 displays a preference for the “inactive” (non-receptor-binding) conformation of arrestins^77,78^, which suggests that this interaction has functions independent of the receptor signaling. Previously we found that parkin also binds arrestin proteins^37^.

We found earlier that parkin dramatically increases the recruitment of Mdm2 to arrestins^37^. Therefore, we asked whether the two ubiquitin ligases, parkin and Mdm2, interact. We showed that they do interact directly using the two purified proteins *in vitro* and demonstrated this interaction in living cells co-expressing these two proteins. The analysis of parkin elements that mediate its interactions with Mdm2 suggests that the C-terminal ring domain (R2) plays the key role. Interestingly, Mdm2 was shown to homo-dimerize via its ring domain^64,79^, which was also implicated in its hetero-dimerization with MdmX (also known as Mdm4), an Mdm2 homolog lacking ubiquitin ligase activity^63,64,79,80^. Both Mdm2 homo- and heterodimers are more efficient ligases than the monomer, possibly due to more effective recruitment of E2 ligases^44,45,79^. A recent structural study suggests that parkin activation by PINK1 phosphorylation at Ser-65 and by the binding of PINK1-phosphorylated ubiquitin results in the rearrangement of parkin domains, with the release of R2, which appears to be a prerequisite of parkin activation^32^. Thus, in view of our finding that R2 domain of parkin mediates Mdm2 binding, it is tempting to speculate that Mdm2 binds parkin via ring-ring interaction and that the release of the R2 induced by Mdm2 binding is the mechanism of parkin activation by Mdm2. Alternatively, the binding of Mdm2 to already released R2 can stabilize active parkin conformation induced by other inputs, such as phosphorylated ubiquitin and/or parkin phosphorylation by PINK1^32^. Our finding that purified Mdm2 increases the activity of purified parkin (Fig. 1B,C) in the absence of PINK1 and phosphorylated ubiquitin is consistent with the first mechanism, but in cells both mechanisms of parkin activation by Mdm2, which are not mutually exclusive, might operate.

Although available evidence strongly suggests a prominent role of the mitochondrial pathology in PD^22,23,72,81^, the exact mechanism involved and the role of parkin in the mitochondrial maintenance in neurons remain obscure. Loss-of-function mutations in parkin are a frequent cause of autosomal recessive familial PD^81,82^ and are commonly found in patients with early onset sporadic PD^4^. Recent structural studies revealed the mechanism of action of several loss-of-function parkin mutations^32^. Compound heterozygous mutations in the parkin gene, which presumably reduce the overall parkin activity, appear to act as a susceptibility factor in the late onset sporadic PD^2–5^. Factors conducive to neurodegeneration, such as stress or dopaminergic toxicity, negatively impact parkin activity^83–86^, also suggesting a role for parkin dysfunction in sporadic PD. Conversely, overexpression of WT parkin affords neuroprotection against a wide variety of insults^87,88^. These data suggest that ligase activity of parkin is necessary for the long-term neuronal survival and that mechanisms regulating its activity are essential for the neuronal health. Several parkin mutations associated with PD are located in the R2 domain, rendering the protein catalytically inactive^87^. We have shown previously^37^ and in this study that catalytically dead parkin with mutations in R2 domain retained the ability to bind Mdm2, suggesting that in patients with hemizygous parkin mutations in R2, catalytically defective mutants could act in a dominant-negative manner, recruiting Mdm2 away from WT parkin generated by the other allele, thereby suppressing its Mdm2-dependent activation. The contribution of this mechanism to PD pathology associated with parkin mutations needs to be tested.

**Figure 8:**
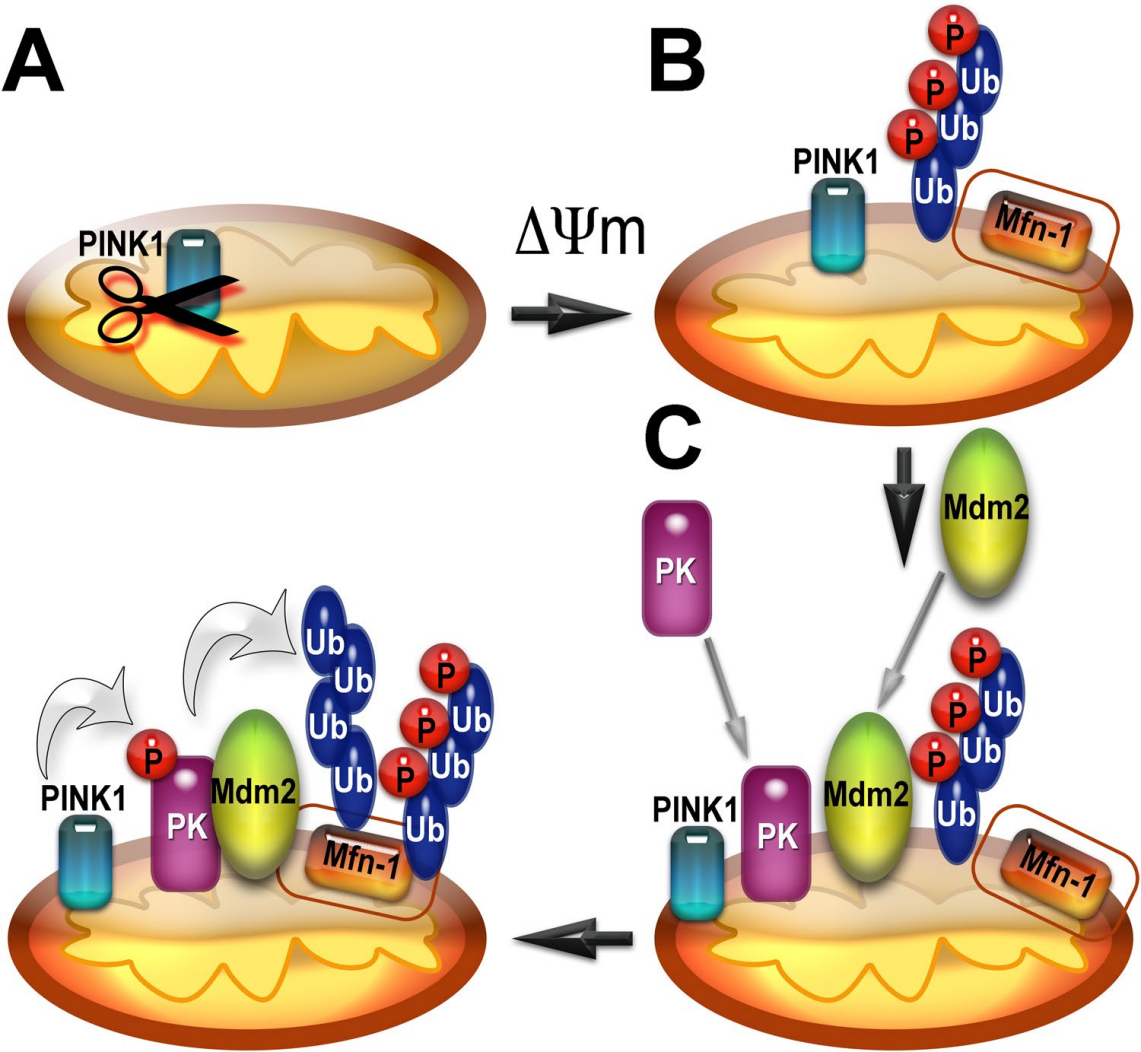
Mdm2 activates parkin and facilitates mitophagy. (**A**) PINK1 is degraded upon binding to healthy mitochondria. (**B**) Upon binding to mitochondria that lost their membrane potential PINK1 is no longer degraded but accumulates on the outer mitochondrial membrane (OMM). It phosphorylates ubiquitin at Ser65. Mitofusin1 (Mfn-1) is a resident protein in OMM in mitochondria. (**C**) Both parkin (PK) and Mdm2 are recruited independently of each other to the mitochondria that lost their membrane potential, although Mdm2 facilitates parkin recruitment. (**D**) PINK1 phosphorylates parkin at Ser-65 in the Ubiquitin-like domain homologous to Ser-65 of ubiquitin. Parkin activated by its phosphorylation, phospho-ubiquitin, and Mdm2 effectively ubiquitinates the OMM proteins including mitofusin1. This leads to degradation of OMM proteins and subsequent mitophagy.

To summarize, here we described a novel mode of parkin regulation via direct binding to another E3 ubiquitin ligase, Mdm2 (Fig. 8). The ability to interact appears to be an inherent characteristic of parkin and Mdm2, as we observed *in vitro* the binding of the two proteins expressed in *E. coli*, where they could not have relevant post-translational modifications. Thus, no special mechanism triggering Mdm2-parkin binding seems to be necessary. Nonetheless, translocation of both proteins to damaged mitochondria would certainly increase their local concentrations in the vicinity of these organelles, thereby promoting their interaction simply by mass action (Fig. 8). Mdm2 binding can increase parkin catalytic activity via disruption of intramolecular self-inhibition in the parkin molecule^27,28^ and/or stabilization of the active conformation with released R2^32^. Importantly, parkin activation by Mdm2 leads to increased parkin-dependent ubiquitination of mitochondrial outer membrane proteins and facilitates mitophagy (Fig. 8), which is believed to be a cytoprotective mechanism. Overall, the interplay between parkin, Mdm2, and its substrate p53 is likely more complex than any single study can reveal (reviewed in^89^). Our data add yet another important piece to this jigsaw puzzle, which still needs to be assembled in its entirety. Considering that Mdm2 and parkin are ubiquitously expressed, our data suggest that the regulation of parkin activity by direct interactions with Mdm2 likely plays an important role in many cell types including neurons. Therefore, our findings have important implications for neuronal survival. As the death of the substantia nigra neurons is the underlying cause of PD, the discovery of parkin activation by direct binding of Mdm2 might pave the way to the development of novel therapeutic strategies.

## Materials and methods

### Antisera and reagents

Rabbit and mouse monoclonal anti-*myc*, mouse anti-HA, and rabbit anti-parkin and rabbit anti-mitofusin1 antibodies were from Cell Signaling Technology (Danvers, MA). Rabbit anti-Tom20 was from Santa Cruz Biotechnology (Santa Cruz, CA). Mouse anti-ubiquitin (P4D1) antibody from Covance (Princeton, NJ) was used to detect ubiquitination. Rabbit and mouse anti-FLAG antibodies were from Sigma Aldrich (St Louis, MO). Rat monoclonal high affinity anti-HA antibody was from Roche Molecular Biochemicals (Indianapolis, IN). E1 and E2 (UbcH7) ubiquitin ligases were purchased from BostonBiochem. CCCP was from Sigma Aldrich. (s)-MG132 was from Cayman Chemical Company (Ann Arbor, MI). Tissue culture media and reagents were from Invitrogen (purchased from ThermoFisher, Waltham, MA), restriction endonucleases from New England Biolabs (Danvers, MA), and all other chemicals were from Sigma Aldrich, unless otherwise specified.

### DNA constructs

DNA constructs were as described previously^37^. Plasmids encoding HA- and myc-tagged WT parkin were generously provided by Dr. Ted Dawson (Johns Hopkins University)^90^. Other mutations were introduced by PCR and confirmed by dideoxy-sequencing. MBP-parkin *E. coli* expression construct (pMal-Parkin) was a generous gift of Dr. Noriyuki Matsuda (Tokyo Metropolitan Institute of Medical Science, Japan)^91^. pMal-MBP was constructed by removing parkin coding sequence and replacing the first parkin codon with a stop codon (between Bam HI and Eco RI sites). GST-Mdm2-100T (Y100-P491) for E. coli expression was a generous gift from Dr. Benjamin Spiller. mKate2-mito expression plasmid was purchased from Axxora (Farmingdale, NY). Four pre-tested human Mdm2 miRNA clones were purchased from Invitrogen. The best clone was selected after testing in HeLa cells. The negative control miRNA was also purchased from Invitrogen. pcDNA3-Mdm2 plasmid (plasmid #16233^92^), YFP-parkin (plasmid #23955^16^), mCherry-parkin (plasmid #23956^16^) and pcDNA3.1-Mfn1-Myc (plasmid #23212^93^) were obtained from Addgene.

### Cell culture and transfection

HEK-293A and HeLa cells were cultured in Dulbecco's modified Eagle medium supplemented with 10% FBS and 1% penicillin-streptomycin in a humidified incubator at 37 °C and 5% CO_2,_ as previously described^37,94^. Lipofectamin 2000 (ThermoFisher) (1:2.5 DNA:lipid) in Opti-MEM was used to transfect cells according to manufacturer's instructions, as described earlier^37^. DNA amounts in each transfection were kept constant by the addition of empty vector. All experiments were conducted 48 h post-transfection.

### Protein purification and *in vitro* binding assays with purified proteins

MBP-parkin and control MBP were purified on maltose column, following described procedure^91,94^.

### GST-Mdm2-100T purification

The pGEX 4T-1 vector containing GST-Mdm2 (Y100-P491) cDNA was transformed into BL21-CodonPlus (DE3)-RIL strain. The cells were grown in LB overnight at 30 °C. GST-Mdm2(Y100-P491) expression was induced by 100 µM isopropyl β-D-thiogalactoside at 30 °C for 5–6 h. The cells from 4 L of culture were pelleted by centrifugation, resuspended in buffer A (150 mM NaCl, 2 mM benzamidine, 0.5 mM PMSF, 10 mM Tris (pH 7.5)), and lysed by freezing and thawing in the presence of lysozyme (3 mg/L), followed by sonication (15 s, 3 times). After centrifugation (12,000 × g, 30 min), the supernatant was loaded onto Glutathione agarose column equilibrated with buffer A. The column was washed with 100 ml of buffer A. GST-tagged proteins were eluted with 400 ml gradient from 0 to 20 mM reduced glutathione. The eluted protein was collected and dialyzed into buffer A. Purified GST-Mdm2-100T was concentrated to around 0.5 mg/ml and stored at −80 °C.

### GST pull-down

The pulldown experiments were performed as we described previously^95^. Purified GST-Mdm2 (Y100-P491) (5 µg) and GST (5 µg) were incubated with 50 µl glutathione–agarose resin (50% slurry, Sigma) at 4 °C overnight with gentle agitation, then purified MBP-parkin or MBP (5 µg) were added and incubated at 4 °C for another 3 h. Suspensions were transferred to centrifuge filters (Ultrafree, 0.65 mm, Millipore) and washed three times with the binding buffer (20 mM Hepes, pH 7.3, 150 mM NaCl). Bound proteins were eluted from glutathione–agarose resin with 100 µl of buffer containing 100 mM reduced glutathione (GSH), 20 mM Hepes-Na, pH 7.3, 150 mM NaCl. The eluted samples were analyzed by SDS-PAGE and Western blot. Samples obtained with GST bound to the resin served as controls for nonspecific binding. Anti-MBP antibody (NEB) was used to detect both MBP and MBP-Parkin in Western blot.

### *In vitro* self-ubiquitination of MBP-parkin

The E3 ubiquitin ligase activity of MBP-parkin was measured according to previous report with minor modifications^96^. Briefly, purified MBP-parkin (20 µg/ml) was incubated in a reaction buffer (50 mM Tris-HCl, pH 8.0, 2 mM dithiothreitol, 5 mM MgCl_2_ and 4 mM ATP) with ubiquitin (50 µg/ml), E1 (1.6 µg/ml), and UbcH7 (20 µg/ml) in the presence of various concentrations of GST-Mdm2-100T (0.02–0.05 µM) at 30 °C for 2 h. The reaction was stopped by the addition of SDS-buffer. The samples were resolved on SDS-PAGE and ubiquitination of MBP-parkin was determined by Western blot. The assays without E1/E2 enzymes served as negative controls.

### *In vivo* ubiquitination assays

To measure self-ubiquitination of parkin, HEK293A cells in 6-well plates were transfected with 2 μg of Flag-Parkin or 0.5 μg of myc-parkin and 0.5 μg of HA-Ubi with or without pcDNA3-Mdm2 plasmid using lipofectamine 2000. Thirty-six hours later, cells were washed with cold PBS and harvested in IP buffer (1% Triton X-100 and Complete Mini protease inhibitor cocktail (Roche Applied Sciences) in PBS). Cell lysates were subjected to immunoprecipitation (IP) with anti-Flag or anti-myc antibody and protein G Sepharose (Millipore). The precipitated proteins were eluted with Laemmli SDS sample buffer and analyzed by Western blot with anti-HA antibody.

### Immunoprecipitation

Immunoprecipitation experiments were performed as we described previously^37^. Indicated cells (HEK293A or HeLa) were scraped off plates, collected by centrifugation in phosphate-buffered saline and resuspended in the immunoprecipitation buffer (IPB) containing 50 mM Tris.HCl, 2 mM EDTA, 250 mM NaCl, 10% (v/v) glycerol, 0.5% NP-40, 20 mM NaF, 1 mM sodium orthovanadate, and 10 mM N-ethylmaleimide. Benzamidine and phenylmethylsulfonyl fluoride (to final concentrations of 2 mM and 1 mM, respectively) were added immediately before use. Cells were lysed at 4°C for 1 h and centrifuged to remove the debris. The supernatant was pre-cleared by incubating with 25–30 μl of Protein G Agarose for 1 h at 4 °C. Target proteins were then immunoprecipitated by incubating the supernatant overnight at 4 °C with appropriate antibodies (1–2 μg per 60 mm Petri dish) and 20–25 μl of Protein G agarose. Beads were washed three times with IPB, and the proteins were eluted by boiling in Laemmli SDS buffer for 5 min,

### Subcellular fractionation

Subcellular fractionation experiments were performed as we described previously^94^. Cells in 100 mm culture dish were harvested by trypsinization and centrifugation at 600 × g for 10 min at 4 °C, washed in phosphate-buffered saline (PBS), resuspended in five volumes of hypotonic buffer (10 mM NaCl, 1.5 mM MgCl_2_, 10 mM Tris-HCl, pH 7.5, protease inhibitors) and incubated 5 min on ice. Cells were homogenized by ten passes through a 26G_5/8_ needle fitted on 1-ml syringe, and sucrose concentration was adjusted to 250 mM with 2 M sucrose. Unbroken cells and nuclei were removed by centrifugation at 1,300 × g for 10 min at 4 °C. The resulting supernatant was centrifuged at 15,000 × g for 15 min at 4 °C to obtain heavy membrane fraction. The supernatant was centrifuged at 100,000 × g for 1 h at 4 °C to obtain cytosolic fraction (S100). Heavy membrane fraction was washed with 10 mM Tris-HCl, pH 7.5, 1 mM EDTA (T10E buffer) containing 250 mM sucrose and solubilized in SDS lysis buffer (1% SDS, 10 mM Tris pH7.4, 1 mM PMSF, protease inhibitors).

### Mdm2 knockdown

Pre-tested Mdm2 miRNA oligonucleotides were purchased from Invitrogen (via ThermoFisher). The antisense miRNA sequence was 5′-AAGCTTGGCACGCCAAACAAA-3′ followed by 19 nucleotide-long loop sequence derived from murine miR-155 followed by sense sequence minus 2 nucleotides. The negative control oligos encoding miRNA that is not homologous to any known mammalian sequence were purchased from Invitrogen. The top and bottom oligonucleotides were annealed and subcloned into the pcDNA6.3 vector, which co-cistronically expressed GFP to label transfected cells.

### Immunofluorescence and microscopy

#### Parkin-dependent mitophagy

For overexpression experiments, HeLa cells in 6 well plates were transfected with GFP or YFP-parkin with or without HA-Mdm2. For knockdown experiments, HeLa cells were transfected with negative control (NegCo) miRNA or Mdm2 miRNA with or without mCherry-parkin. Cells were plated on fibronectin-coated chamber slides (Lab-Tek 177399 from ThermoFisher) and fixed with methanol at −20 °C for 2 min, then permeabilized by 0.5% Triton X-100/PBS for 10 min. After blocking with 5% BSA/PBS for 1 hr with gentle rocking, the cells were incubated with primary antibodies overnight at 4°C in PBS/2%BSA/0.03%Triton X-100. In the Mdm2 overexpression experiments rabbit anti-Tom20 antibodies (Santa Cruz Biotechnology; FL-145; 1:50 dilution) were used to visualize the mitochondria combined with mouse anti-HA antibody (Cell Signaling; #2367; 1:100 dilution) to detect Mdm2 [in the CFP control plates, mouse anti-GFP antibody (Clontech, JL-8, 1:500) was used instead]. Dylight 405-conjuated affinipure Donkey anti-rabbit (Jackson Immunoresearch Laboratories; 1:200) and AlexaFluor 594 chicken anti-mouse (Invitrogen; 1:200) were used as secondary antibodies. In the experiment with Mdm2 miRNA knockdown, a combination of mouse anti-GFP (Clontech, JL-8, 1:500), rabbit anti-Tom 20 (Santa Cruz Biotechnology; FL-145; 1:50 dilution), and chicken anti-mCherry antibodies (Novusbio, 1:500) was used to simultaneously label the cells for miRNA (NegCo or Mdm2), mCherry-parkin, and the mitochondria. Anti-rabbit biotinylated antibody followed by Alexa 405 streptavidin, donkey anti-chicken Alexa 594, and goat anti-mouse Alexa 488 secondary antibodies were used. Coverslips were mounted onto microscope slides using Vectashield mounting medium from Vector Laboratories. Samples were imaged using a LSM710 confocal microscope in green, red, and blue channels. The original unprocessed confocal images were analyzed using ImageJ (NIH). The cells expressing GFP (control), YFP-parkin or YFP-parkin plus HA-Mdm2 (in the overexpression experiment) or cell expressing negative control miRNA + GFP or Mdm2 miRNA + GFP with or without mCherry-parkin (in the knockdown experiment) were classified as having normal mitochondria, fragmented mitochondria, or no mitochondria by an observer blind to the experimental conditions. Only Tom20 images (blue) were used for scoring, with the cells to be scored outlined, so the “blind” observer did not know which protein(s) these cells expressed. For presentation purposes, contrast was adjusted for each color to better show double- or triple-labeled cells.

#### For Mdm2 recruitment

HeLa cells were transfected with Mdm2-GFP and mKate-mito, plated on Mattek dishes, treated with 10 μM CCCP for 1, 2, or 4 h and imaged live on Nikon TE2000-E microscope with 40X oil objective. In case of washout, HeLa cells expressing Mdm2-GFP and mKate-mito were treated with 10 μM CCCP for 2 h, then the medium was replaced and the cells were incubated for ~12 h in CCCP-free medium.

### Statistical analysis

StatView (SAS Institute, Cary, NC) software was used for statistical analysis of quantitative data. The data were analyzed by one-way analysis of variance (ANOVA) followed by Bonferroni/Dunn post hoc comparison of means with correction for multiple comparisons. In all cases, p<0.05 was considered significant. Correlation between Mdm2 expression and parkin activity was analyzed by ANCOVA with Protein as main factor followed by Bonferroni/Dunn post hoc comparison of means with correction for multiple comparisons.

## Supplementary information

Supplementary Information.

## References

[1] Kitada T (1998). Mutations in the parkin gene cause autosomal recessive juvenile parkinsonism. Nature.

[2] Farrer M (2001). Lewy bodies and parkinsonism in families with parkin mutations. Ann. Neurol..

[3] Khan NL (2005). Dopaminergic dysfunction in unrelated, asymptomatic carriers of a single parkin mutation. Neurology.

[4] Lücking CB (2000). Association between early-onset Parkinson's disease and mutations in the Parkin gene. N. Engl. J. Med..

[5] Pramstaller PP (2005). Lewy body Parkinson's disease in a large pedigree with 77 Parkin mutation carriers. Ann. Neurol..

[6] Shimura H (2000). Familial Parkinson disease gene product, parkin, is a ubiquitin-protein ligase. Nat. Genet..

[7] Savitt JM, Dawson VL, Dawson TM (2006). Diagnosis and treatment of Parkinson disease: molecules to medicine. J. Clin. Invest..

[8] Winklhofer KF (2014). Parkin and mitochondrial quality control: toward assembling the puzzle. Trends Cell Biol..

[9] Ashrafi G, Schwarz TL (2013). The pathways of mitophagy for quality control and clearance of mitochondria. Cell Death Differ..

[10] McWilliams TG, Muqit MM (2017). PINK1 and Parkin: emerging themes in mitochondrial homeostasis. Curr. Opin. Cell Biol..

[11] Valente EM (2004). Hereditary early-onset Parkinson's disease caused by mutations in PINK1. Science.

[12] Vives-Bauza C (2010). PINK1-dependent recruitment of Parkin to mitochondria in mitophagy. Proc. Natl. Acad. Sci. USA.

[13] Matsuda N (2010). PINK1 stabilized by mitochondrial depolarization recruits Parkin to damaged mitochondria and activates latent Parkin for mitophagy. J. Cell Biol..

[14] Kane LA (2014). PINK1 phosphorylates ubiquitin to activate Parkin E3 ubiquitin ligase activity. J. Cell Biol..

[15] Sha D, Chin LS, Li L (2010). Phosphorylation of parkin by Parkinson disease-linked kinase PINK1 activates parkin E3 ligase function and NF-kappaB signaling. Hum. Mol. Genet..

[16] Narendra D, Tanaka A, Suen DF, Youle RJ (2008). Parkin is recruited selectively to impaired mitochondria and promotes their autophagy. J. Cell Biol..

[17] Gegg ME (2010). Mitofusin 1 and mitofusin 2 are ubiquitinated in a PINK1/parkin-dependent manner upon induction of mitophagy. Hum. Mol. Genet..

[18] Tanaka A (2010). Proteasome and p97 mediate mitophagy and degradation of mitofusins induced by Parkin. J. Cell Biol..

[19] Yoshii SR, Kishi C, Ishihara N, Mizushima N (2011). Parkin mediates proteasome-dependent protein degradation and rupture of the outer mitochondrial membrane. J. Biol. Chem..

[20] Hauser DN, Hastings TG (2013). Mitochondrial dysfunction and oxidative stress in Parkinson's disease and monogenic parkinsonism. Neurobiol. Dis..

[21] de Vries RL, Przedborski S (2013). Mitophagy and Parkinson's disease: be eaten to stay healthy. Mol. Cell. Neurosci..

[22] Scott L, Dawson VL, Dawson TM (2017). Trumping neurodegeneration: Targeting common pathways regulated by autosomal recessive Parkinson's disease genes. Exp. Neurol..

[23] Zhang, C.-W., Hang, L., Yao, T.-P. & Lim, K.-L. Parkin Regulation and Neurodegenerative Disorders. *Front. Aging Neurosci*. **7**, 10.3389/fnagi.2015.00248 (2016). 10.3389/fnagi.2015.00248 PMC4709595 26793099

[24] Van Laar VS (2011). Bioenergetics of neurons inhibit the translocation response of Parkin following rapid mitochondrial depolarization. Hum. Mol. Genet..

[25] Sterky FH, Lee S, Wibom R, Olson L, Larsson NG (2011). Impaired mitochondrial transport and Parkin-independent degeneration of respiratory chain-deficient dopamine neurons *in vivo*. Proc. Natl. Acad. Sci. USA.

[26] Grenier K, McLelland GL, Fon EA (2013). Parkin- and PINK1-dependent mitophagy in neurons: Will the real pathway please stand up?. Front. Neurol..

[27] Trempe JF (2013). Structure of parkin reveals mechanisms for ubiquitin ligase activation. Science.

[28] Wauer T, Komander D (2013). Structure of the human Parkin ligase domain in an autoinhibited state. EMBO J..

[29] Chaugule VK (2011). Autoregulation of Parkin activity through its ubiquitin-like domain. EMBO J..

[30] Riley BE (2013). Structure and function of Parkin E3 ubiquitin ligase reveals aspects of RING and HECT ligases. Nat. Commun..

[31] Iguchi M (2013). Parkin-catalyzed ubiquitin-ester transfer is triggered by PINK1-dependent phosphorylation. J. Biol. Chem..

[32] Gladkova C, Maslen SL, Skehel JM, Komander D (2018). Mechanism of parkin activation by PINK1. Nature.

[33] Imai Y (2002). CHIP is associated with Parkin, a gene responsible for familial Parkinson's disease, and enhances its ubiquitin ligase activity. Mol. Cell.

[34] Staropoli JF (2003). Parkin is a component of an SCF-like ubiquitin ligase complex and protects postmitotic neurons from kainate excitotoxicity. Neuron.

[35] Kalia SK (2004). BAG5 inhibits parkin and enhances dopaminergic neuron degeneration. Neuron.

[36] Walden H, Martinez-Torres RJ (2012). Regulation of Parkin E3 ubiquitin ligase activity. Cell Mol. Life Sci..

[37] Ahmed MR (2011). Ubiquitin ligase parkin promotes Mdm2-arrestin interaction but inhibits arrestin ubiquitination. Biochemistry.

[38] Gurevich VV, Gurevich EV (2006). The structural basis of arrestin-mediated regulation of G-protein-coupled receptors. Pharmacol. Ther..

[39] Gurevich EV, Gurevich VV (2006). Arrestins: ubiquitous regulators of cellular signaling pathways. Genome Biol..

[40] DeWire SM, Ahn S, Lefkowitz RJ, Shenoy SK (2007). Beta-arrestins and cell signaling. Ann. Rev. Physiol..

[41] Gurevich VV, Gurevich EV (2015). Analyzing the roles of multi-functional proteins in cells: The case of arrestins and GRKs. Crit. Rev. Biochem. Mol. Biol..

[42] Perry NA (2019). Arrestin-3 scaffolding of the JNK3 cascade suggests a mechanism for signal amplification. Proc. Natl. Acad. Sci. USA.

[43] Fang S, Jensen JP, Ludwig RL, Vousden KH, Weissman AM (2000). Mdm2 is a RING finger-dependent ubiquitin protein ligase for itself and p53. J. Biol. Chem..

[44] Wade M, Li YC, Wahl GM (2013). MDM2, MDMX and p53 in oncogenesis and cancer therapy. Nat. Rev. Cancer.

[45] Wade M, Wang YV, Wahl GM (2010). The p53 orchestra: Mdm2 and Mdmx set the tone. Trends Cell Biol..

[46] Momand J, Zambetti GP, Olson DC, George D, Levine AJ (1992). The mdm-2 oncogene product forms a complex with the p53 protein and inhibits p53-mediated transactivation. Cell.

[47] Kussie PH (1996). Structure of the MDM2 oncoprotein bound to the p53 tumor suppressor transactivation domain. Science.

[48] Hoshino A (2013). Cytosolic p53 inhibits Parkin-mediated mitophagy and promotes mitochondrial dysfunction in the mouse heart. Nat. Commun..

[49] Song YM (2016). Metformin Restores Parkin-Mediated Mitophagy, Suppressed by Cytosolic p53. Int. J. Mol. Sci..

[50] Zheng R (2015). TAT-ODD-p53 enhances the radiosensitivity of hypoxic breast cancer cells by inhibiting Parkin-mediated mitophagy. Oncotarget.

[51] Arena G (2018). Mitochondrial MDM2 regulates respiratory complex I activity independently of p53. Mol. Cell.

[52] Shenoy SK, McDonald PH, Kohout TA, Lefkowitz RJ (2001). Regulation of receptor fate by ubiquitination of activated beta 2-adrenergic receptor and beta-arrestin. Science.

[53] Shenoy SK (2009). Beta-arrestin-dependent signaling and trafficking of 7-transmembrane receptors is reciprocally regulated by the deubiquitinase USP33 and the E3 ligase Mdm2. Proc. Natl. Acad. Sci. USA.

[54] Wang P (2003). Beta -Arrestin 2 functions as a G-protein-coupled receptor-activated regulator of oncoprotein Mdm2. J. Biol. Chem..

[55] Boularan C (2007). Beta-arrestin 2 oligomerization controls the Mdm2-dependent inhibition of p53. Proc. Natl. Acad. Sci. USA.

[56] Gama V (2014). The E3 ligase PARC mediates the degradation of cytosolic cytochrome c to promote survival in neurons and cancer cells. Sci. Signal..

[57] Sriram SR (2005). Familial-associated mutations differentially disrupt the solubility, localization, binding and ubiquitination properties of parkin. Hum. Mol. Genet..

[58] Hampe C, Ardila-Osorio H, Fournier M, Brice A, Corti O (2006). Biochemical analysis of Parkinson's disease-causing variants of Parkin, an E3 ubiquitin-protein ligase with monoubiquitination capacity. Hum. Mol. Genet..

[59] Saville MK (2004). Regulation of p53 by the ubiquitin-conjugating enzymes UbcH5B/C *in vivo*. J. Biol. Chem..

[60] van Wijk SJ (2009). A comprehensive framework of E2-RING E3 interactions of the human ubiquitin-proteasome system. Mol. Syst. Biol..

[61] Lazarou M (2013). PINK1 drives Parkin self-association and HECT-like E3 activity upstream of mitochondrial binding. J. Cell Biol..

[62] Wenzel DM, Lissounov A, Brzovic PS, Klevit RE (2011). UBCH7 reactivity profile reveals parkin and HHARI to be RING/HECT hybrids. Nature.

[63] Linke K (2008). Structure of the MDM2/MDMX RING domain heterodimer reveals dimerization is required for their ubiquitylation in trans. Cell Death Differ..

[64] Kawai H, Lopez-Pajares V, Kim MM, Wiederschain D, Yuan ZM (2007). RING domain-mediated interaction is a requirement for MDM2's E3 ligase activity. Cancer Res..

[65] Ding WX (2012). Parkin and mitofusins reciprocally regulate mitophagy and mitochondrial spheroid formation. J. Biol. Chem..

[66] Hauck L (2017). Cardiac-specific ablation of the E3 ubiquitin ligase Mdm2 leads to oxidative stress, broad mitochondrial deficiency and early death. PLoS One.

[67] Shin JH (2011). PARIS (ZNF746) repression of PGC-1α contributes to neurodegeneration in Parkinson's disease. Cell.

[68] Stevens DA (2015). Parkin loss leads to PARIS-dependent declines in mitochondrial mass and respiration. Proceedings of the National Academy of Sciences.

[69] Bernardini JP, Lazarou M, Dewson G (2017). Parkin and mitophagy in cancer. Oncogene.

[70] Wahabi K, Perwez A, Rizvi MA (2018). Parkin in Parkinson's Disease and Cancer: a Double-Edged Sword. Molecular Neurobiology.

[71] Escobar-Henriques, M. & Joaquim, M. Mitofusins: Disease Gatekeepers and Hubs in Mitochondrial Quality Control by E3 Ligases. *Frontiers in Physiology* **10**, 10.3389/fphys.2019.00517 (2019). 10.3389/fphys.2019.00517 PMC6533591 31156446

[72] Scarffe LA, Stevens DA, Dawson VL, Dawson TM (2014). Parkin and PINK1: much more than mitophagy. Trends Neurosci..

[73] Chen Y, Dorn GWn (2013). PINK1-phosphorylated mitofusin 2 is a Parkin receptor for culling damaged mitochondria. Science.

[74] Jung YY (2017). Loss of Parkin reduces inflammatory arthritis by inhibiting p53 degradation. Redox Biol..

[75] Kobet E, Zeng X, Zhu Y, Keller D, Lu H (2000). MDM2 inhibits p300-mediated p53 acetylation and activation by forming a ternary complex with the two proteins. Proc. Natl. Acad. Sci. USA.

[76] Montes de Oca Luna R, Wagner DS, Lozano G (1995). Rescue of early embryonic lethality in mdm2-deficient mice by deletion of p53. Nature.

[77] Hanson SM (2007). Arrestin mobilizes signaling proteins to the cytoskeleton and redirects their activity. J. Mol. Biol..

[78] Song X, Raman D, Gurevich EV, Vishnivetskiy SA, Gurevich VV (2006). Visual and both non-visual arrestins in their “inactive” conformation bind JNK3 and Mdm2 and relocalize them from the nucleus to the cytoplasm. J. Biol. Chem..

[79] Poyurovsky MV (2007). The Mdm2 RING domain C-terminus is required for supramolecular assembly and ubiquitin ligase activity. EMBO J..

[80] Uldrijan S, Pannekoek WJ, Vousden KH (2007). An essential function of the extreme C-terminus of MDM2 can be provided by MDMX. EMBO J..

[81] Corti O, Lesage S, Brice A (2011). What genetics tells us about the causes and mechanisms of Parkinson's disease. Physiol. Rev..

[82] Singleton AB, Farrer MJ, Bonifati V (2013). The genetics of Parkinson's disease: progress and therapeutic implications. Mov. Disord..

[83] Chung KK (2004). S-nitrosylation of parkin regulates ubiquitination and compromises parkin's protective function. Science.

[84] LaVoie MJ, Ostaszewski BL, Weihofen A, Schlossmacher MG, Selkoe DJ (2005). Dopamine covalently modifies and functionally inactivates parkin. Nature Med..

[85] Winklhofer KF, Henn IH, Kay-Jackson PC, Heller U, Tatzelt J (2003). Inactivation of parkin by oxidative stress and C-terminal truncations: a protective role of molecular chaperones. J. Biol. Chem..

[86] Wong ES (2007). Relative sensitivity of parkin and other cysteine-containing enzymes to stress-induced solubility alterations. J. Biol. Chem..

[87] Moore DJ, West AB, Dawson VL, Dawson TM (2005). Molecular pathophysiology of Parkinson's disease. Annu. Rev. Neurosci..

[88] Dai Y, Hu X, Sun X (2018). Overexpression of parkin protects retinal ganglion cells in experimental glaucoma. Cell. Death. Dis..

[89] Checler F (2014). & Alves da Costa, C. Interplay between parkin and p53 governs a physiological homeostasis that is disrupted in Parkinson's disease and cerebral cancer. Neurodegener. Dis..

[90] Moore DJ, West AB, Dikeman DA, Dawson VL, Dawson TM (2008). Parkin mediates the degradation-independent ubiquitination of Hsp70. J. Neurochem..

[91] Matsuda N (2006). Diverse effects of pathogenic mutations of Parkin that catalyze multiple monoubiquitylation *in vitro*. J. Biol. Chem..

[92] Zhou BP (2001). HER-2/neu induces p53 ubiquitination via Akt-mediated MDM2 phosphorylation. Nat. Cell. Biol..

[93] Chen H (2003). Mitofusins Mfn1 and Mfn2 coordinately regulate mitochondrial fusion and are essential for embryonic development. J. Cell Biol..

[94] Kook S (2014). Caspase-cleaved arrestin-2 and BID cooperatively facilitate cytochrome C release and cell death. Cell Death Differ..

[95] Zhan X (2016). Peptide mini-scaffold facilitates JNK3 activation in cells. Sci. Rep..

[96] Yamamoto A (2005). Parkin phosphorylation and modulation of its E3 ubiquitin ligase activity. J. Biol. Chem..

