# Supplementary material for: Mdm2 enhances ligase activity of parkin and facilitates mitophagy
Source: Sci Rep. 2020 Mar 19;10:5028. doi: 10.1038/s41598-020-61796-4 (PMC7081349; doi:10.1038/s41598-020-61796-4)
Supplement: Supplementary file 1 — Supplementary Information. [file 41598_2020_61796_MOESM1_ESM.docx]

**Mdm2 enhances ligase activity of parkin and facilitates mitophagy**

Seunghyi Kook, Xuanzhi Zhan, Kimberly Thibeault, Mohamed R. Ahmed, Vsevolod V. Gurevich, and Eugenia V. Gurevich

Department of Pharmacology, Vanderbilt University, Nashville, TN 37232

**Uncropped Western blot images**

**FIGURE 1**

**
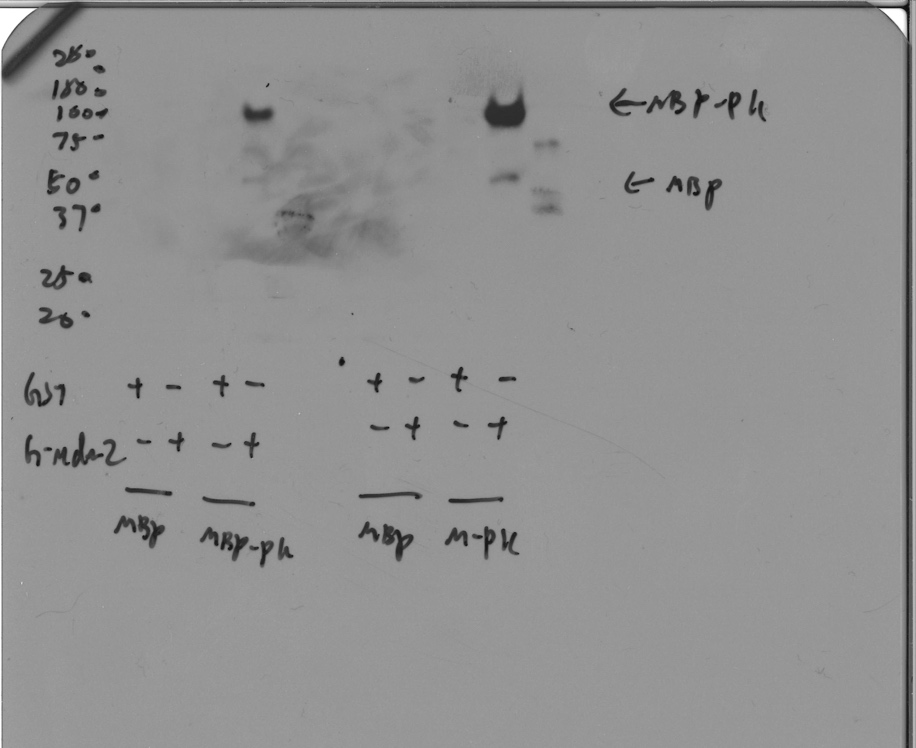
**

**Fig 1A:** GST pulldown; anti-MBP blot; two different loads.


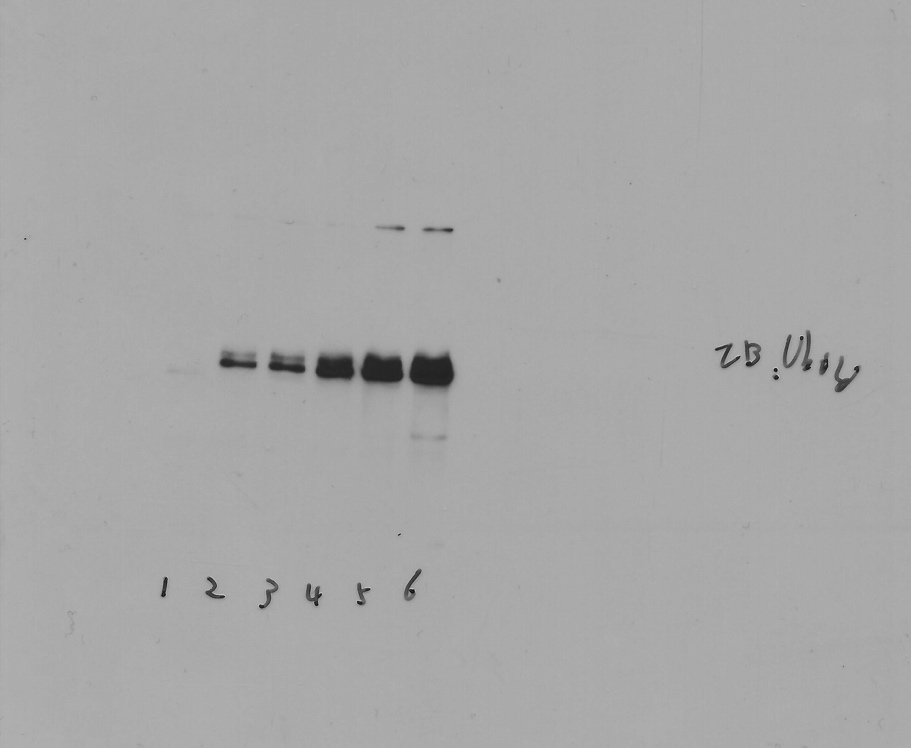


**Fig 1B**: Ubiquitin blot


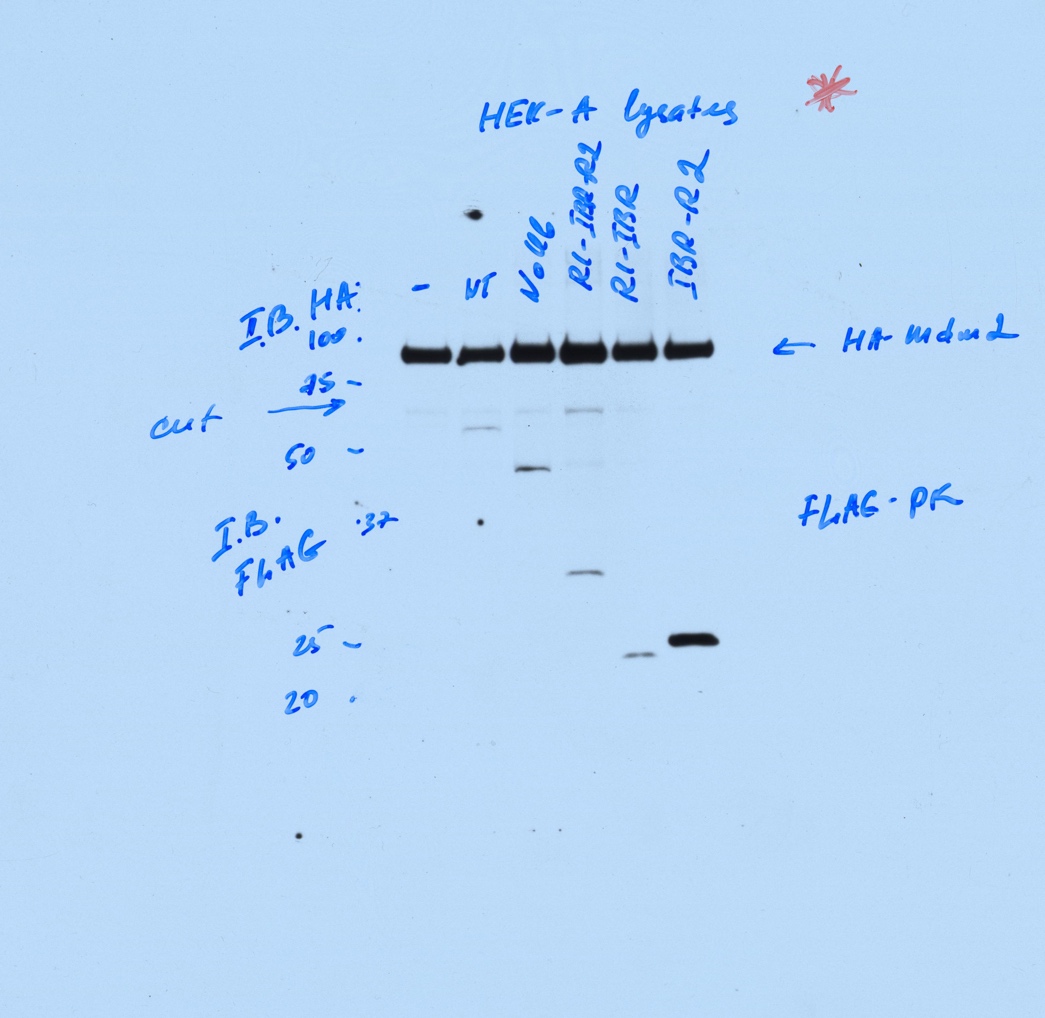


**Fig 1E left panel:** the blot was cut horizontally and the upper portion probed with anti-AH antibody (for HA-Mdm2) and the lower - with anti-FLAG (for FLAG-Parkin fragments).


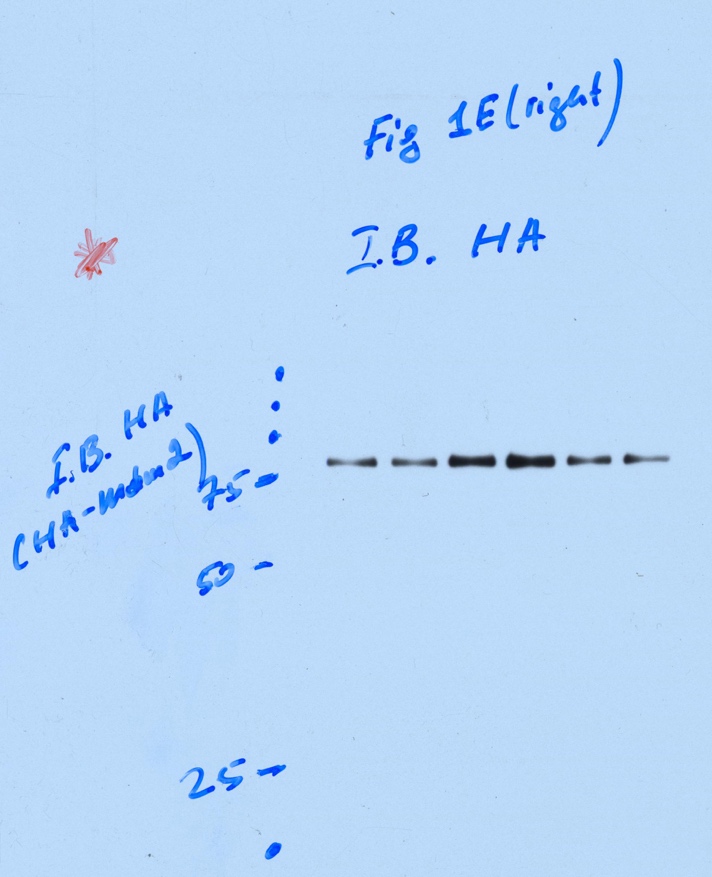


**Fig 1E right panel:** IB HA (H-Mdm2).

**FIGURE 2**


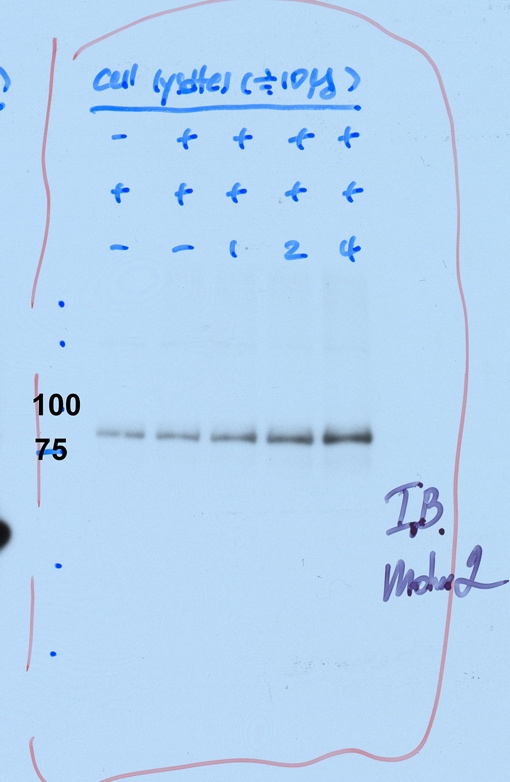


**Fig 2A left panel:** HEK cell lysates Mdm2


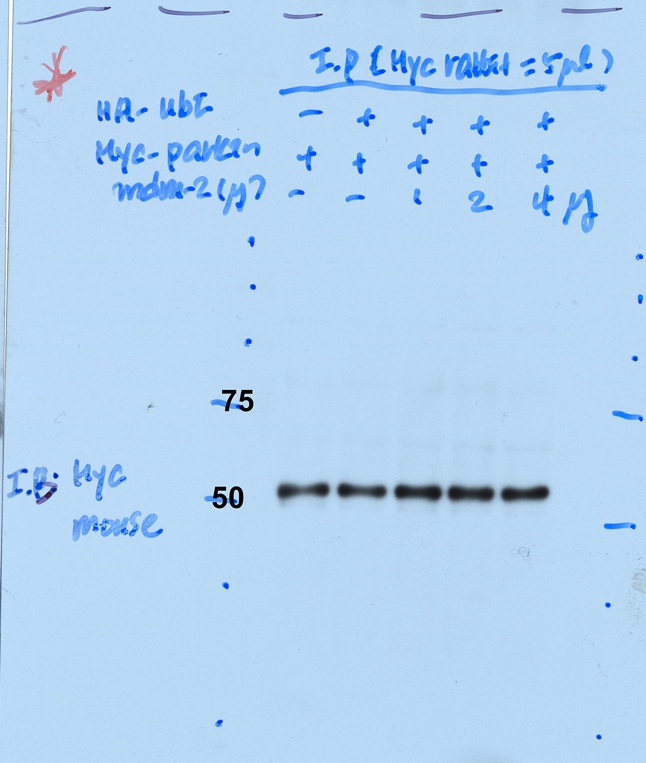


**Fig 2A right panel:** IP myc IB myc (myc-parkin)


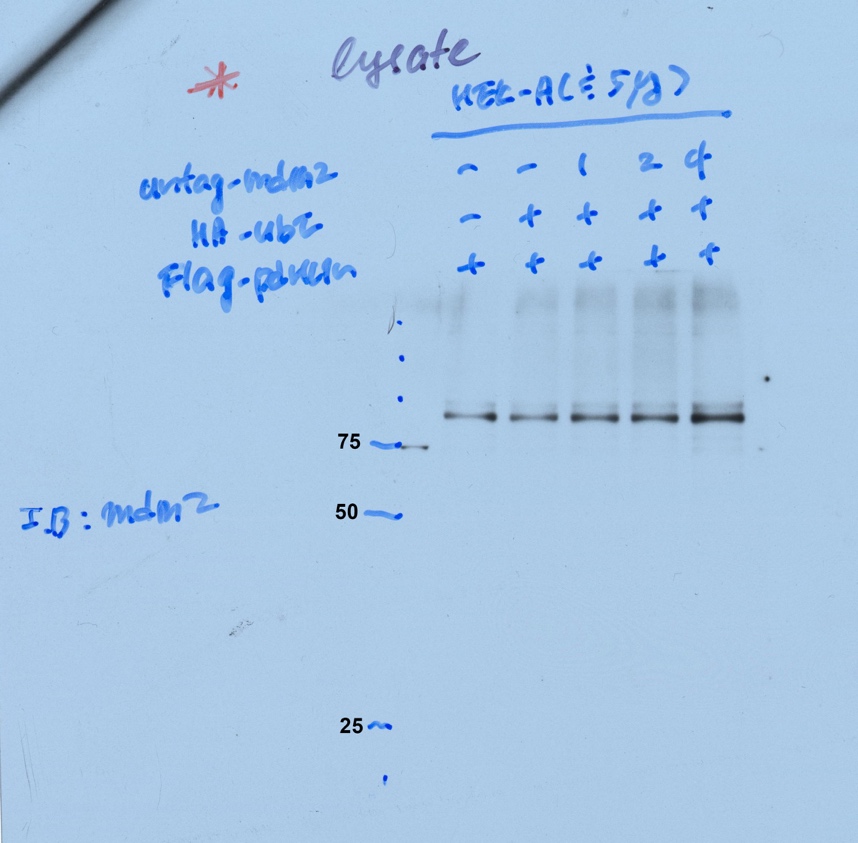


**Fig 2B left panel:** lysates Mdm2


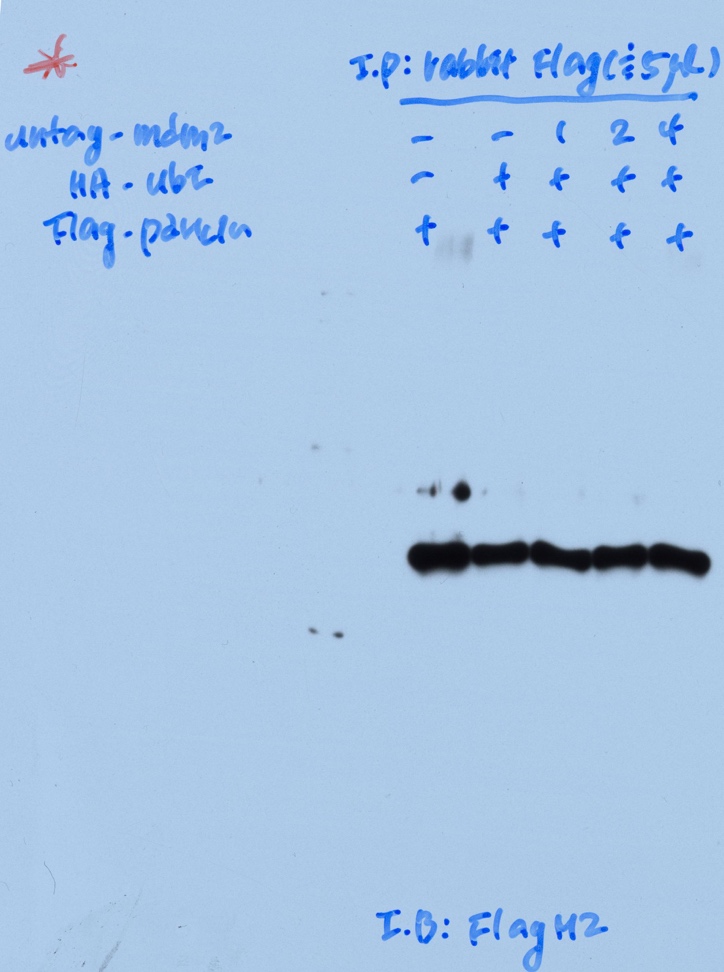


**Fig 2B right panel:** IP FLAG IB FLAG

**FIGURE 3**


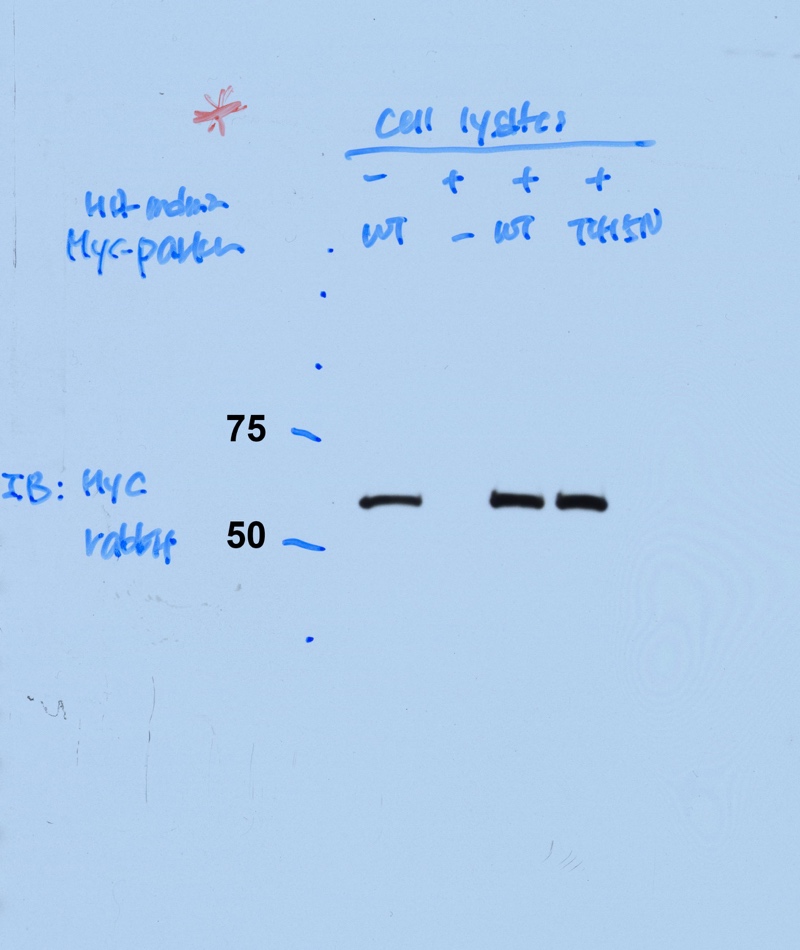


**Fig 3A right panel:** IB myc (myc-parkin) cell lysates


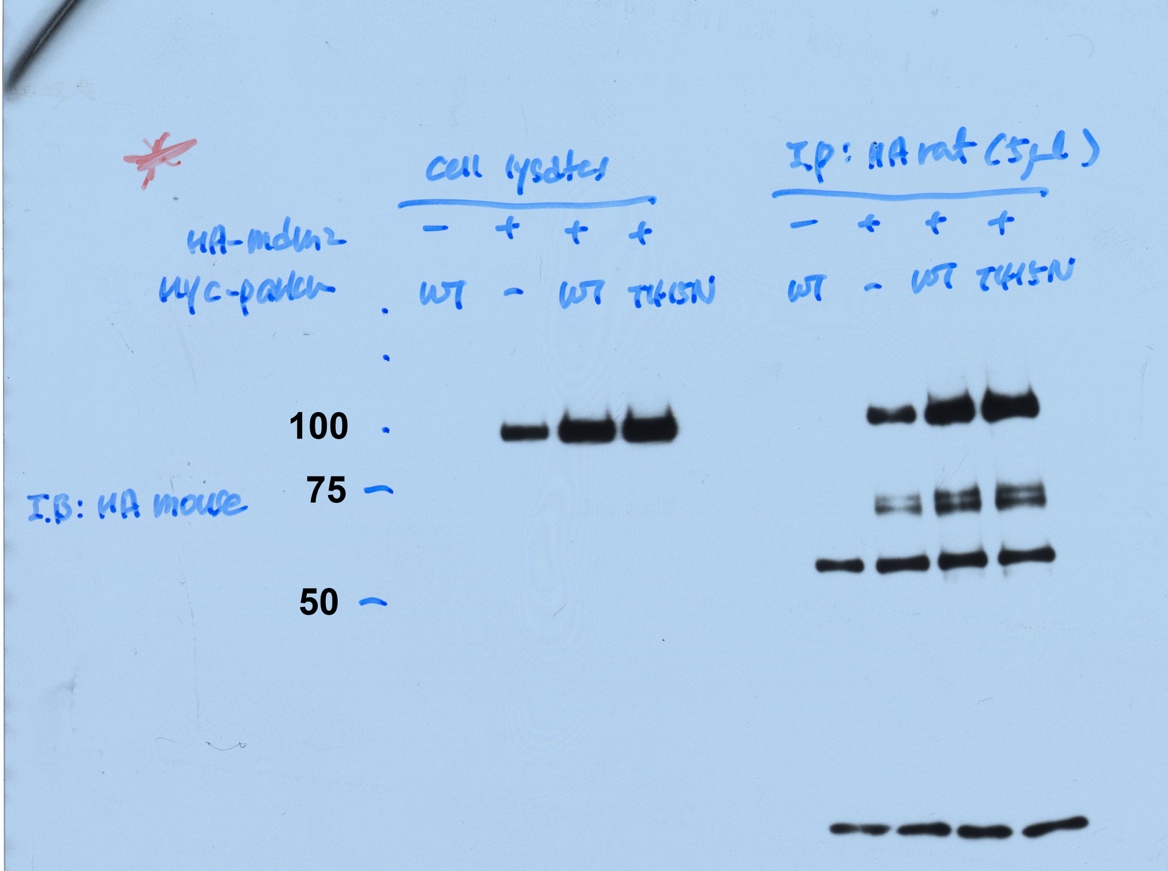


**Fig 3A:** IB HA cell lysates (right panel in Fig 3A) and IP HA (left panel in Fig 3A)


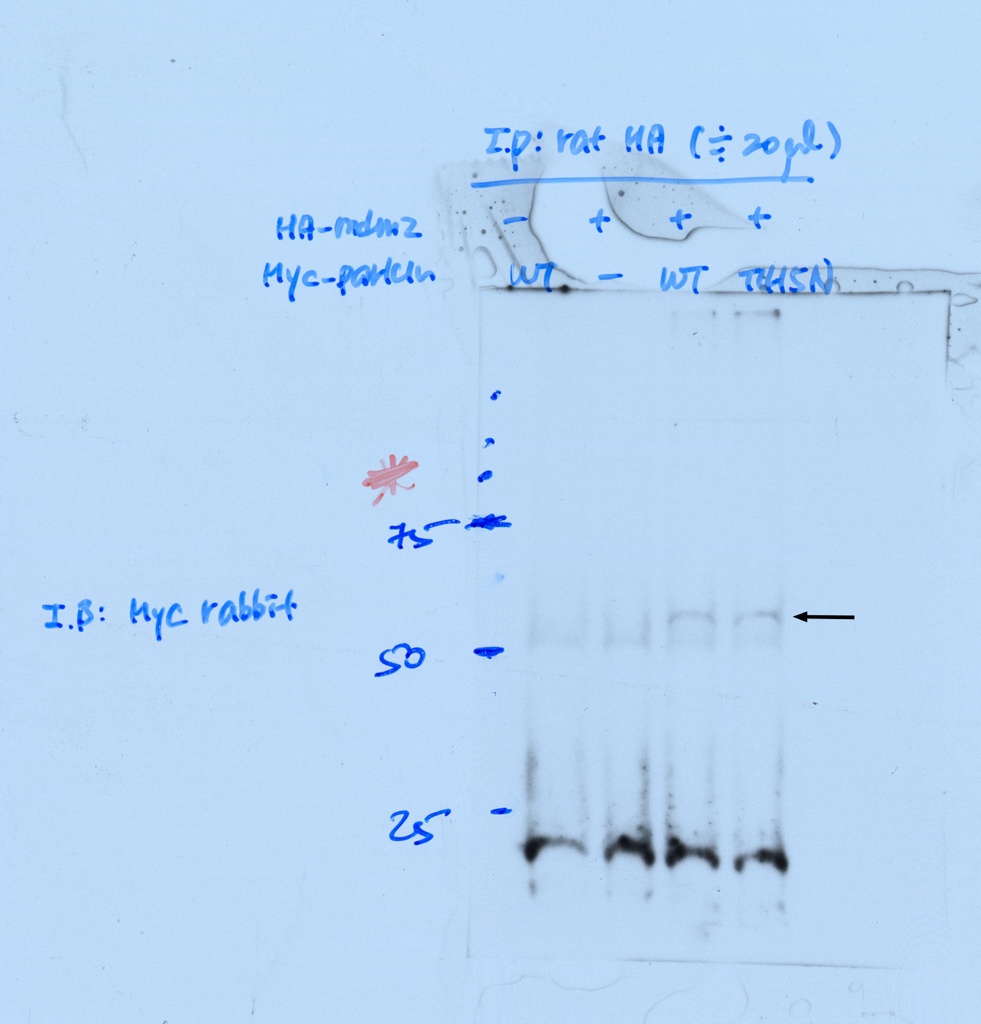


**Fig 3A left panel:** IP HA IB myc


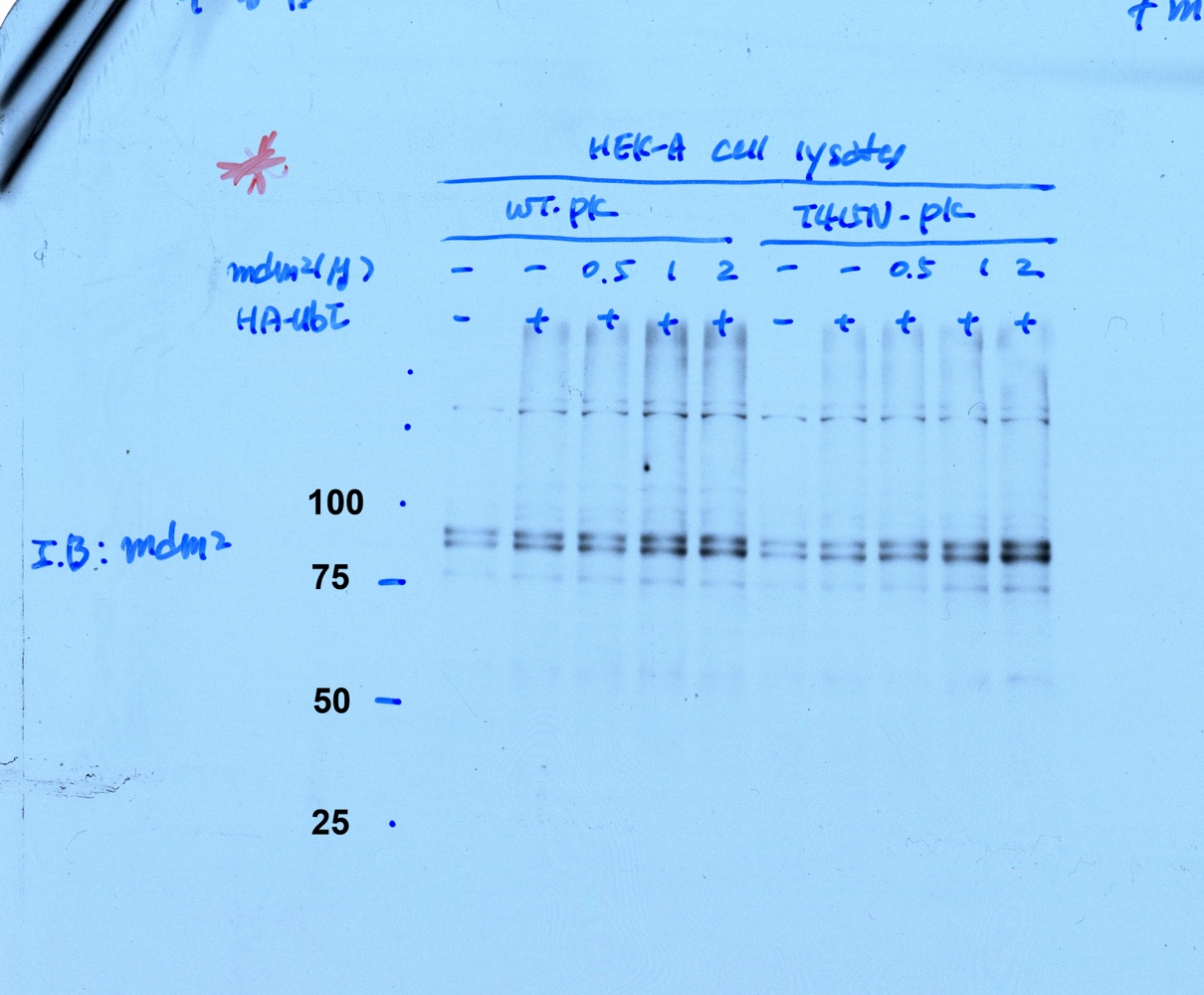


**Fig 3B upper panel:** Mdm2 cell lysates


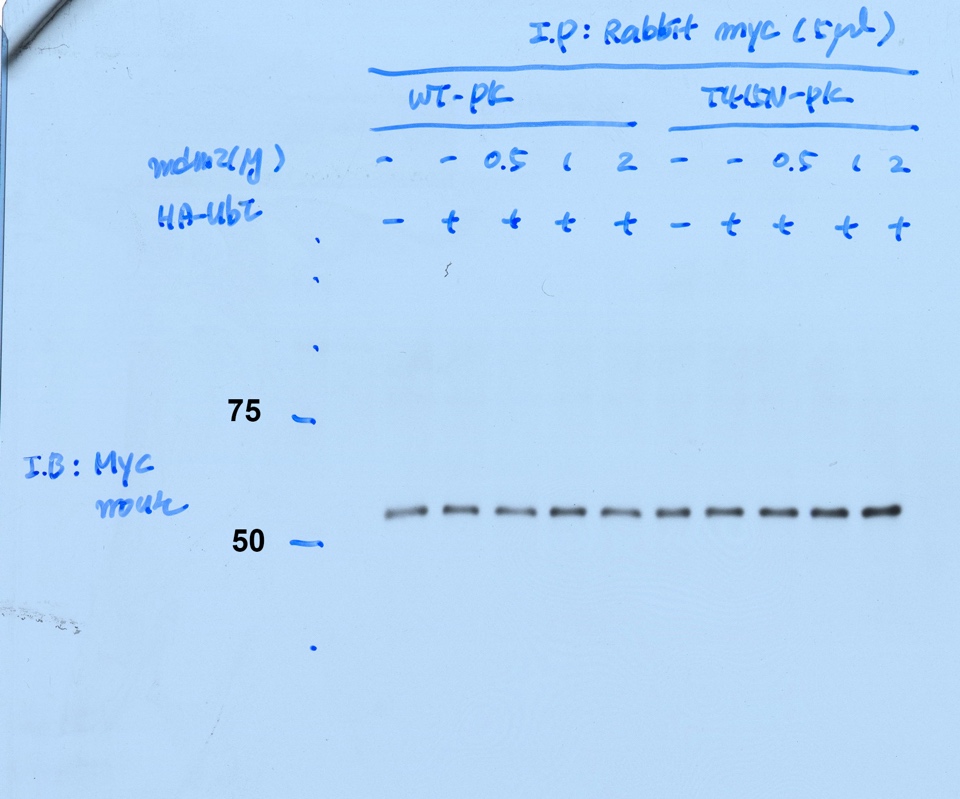


**Fig 3B lower panel:** IP myc IB myc (myc-parkin)

**FIGURE 4**

**
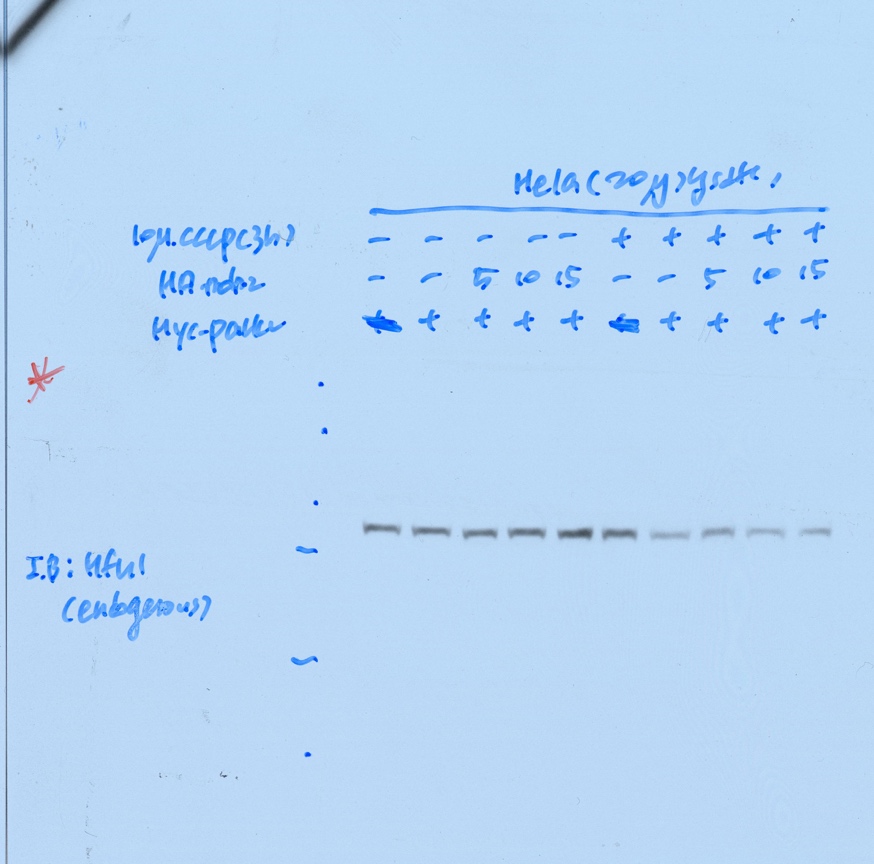
**

**Fig 4A:** IB Mfs-1 short exposure


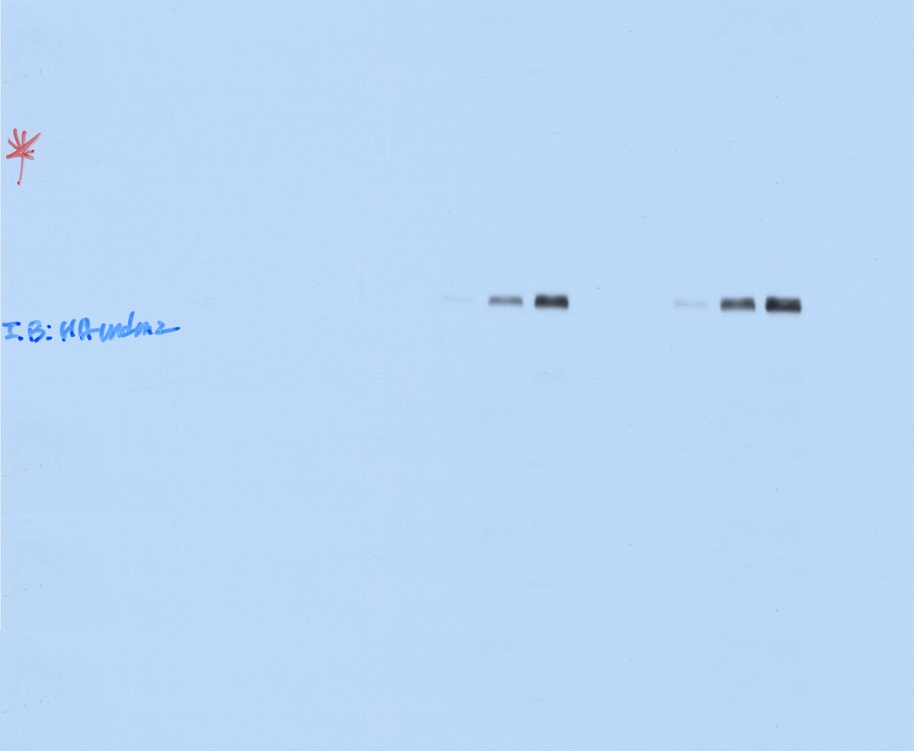


**Fig 4A:** IB HA - HA-Mdm2


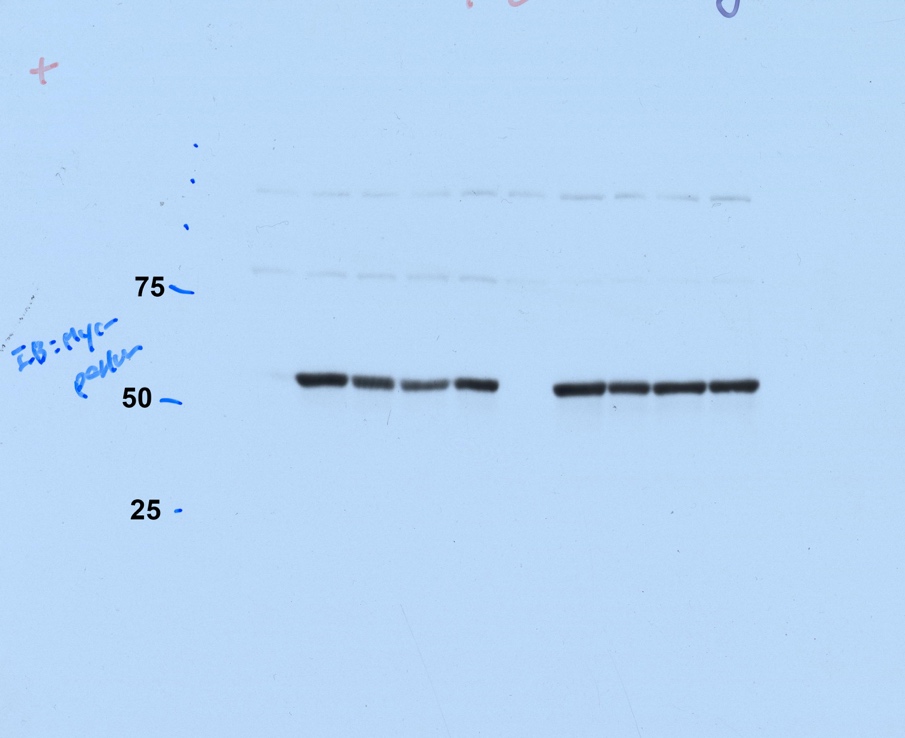


**Fig 4A:** IB myc - myc-parkin


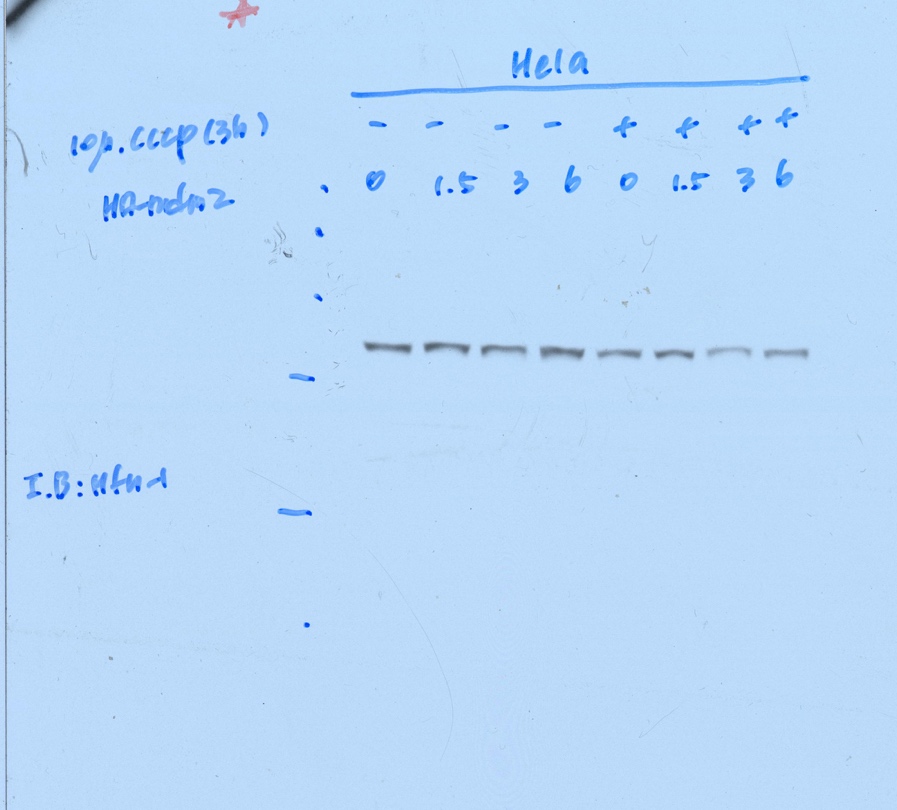


**Fig 4B:** IB Mfs-1 short exposure

**Fig 4D upper panel:** IB myc (myc-Mfs-1)


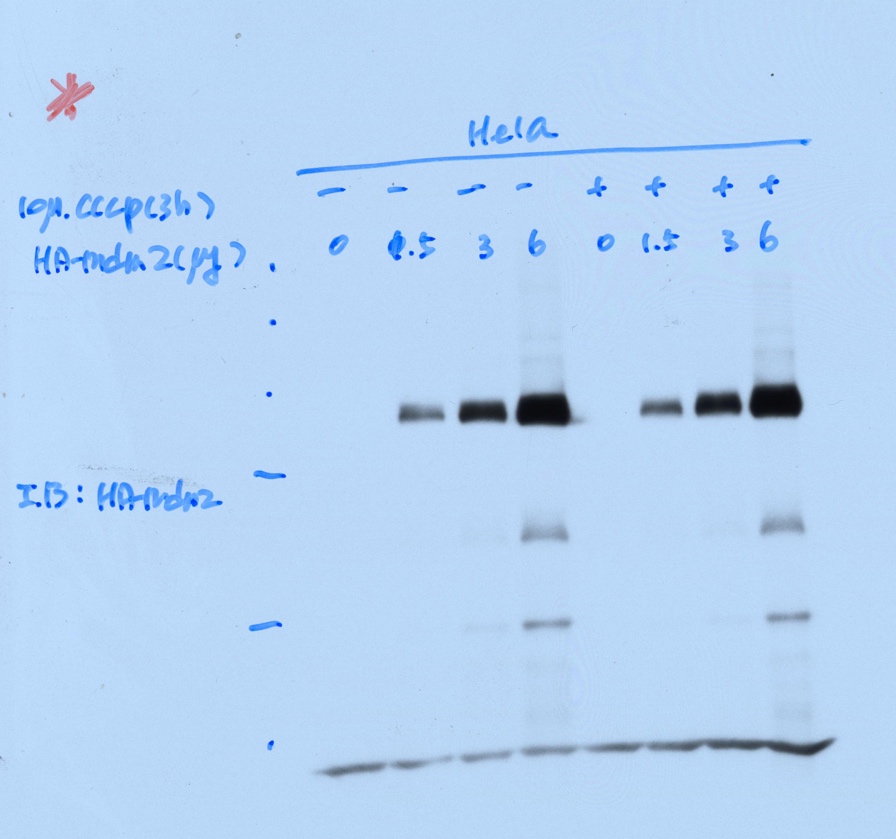


**Fig 4B:** IB HA - HA-Mdm2


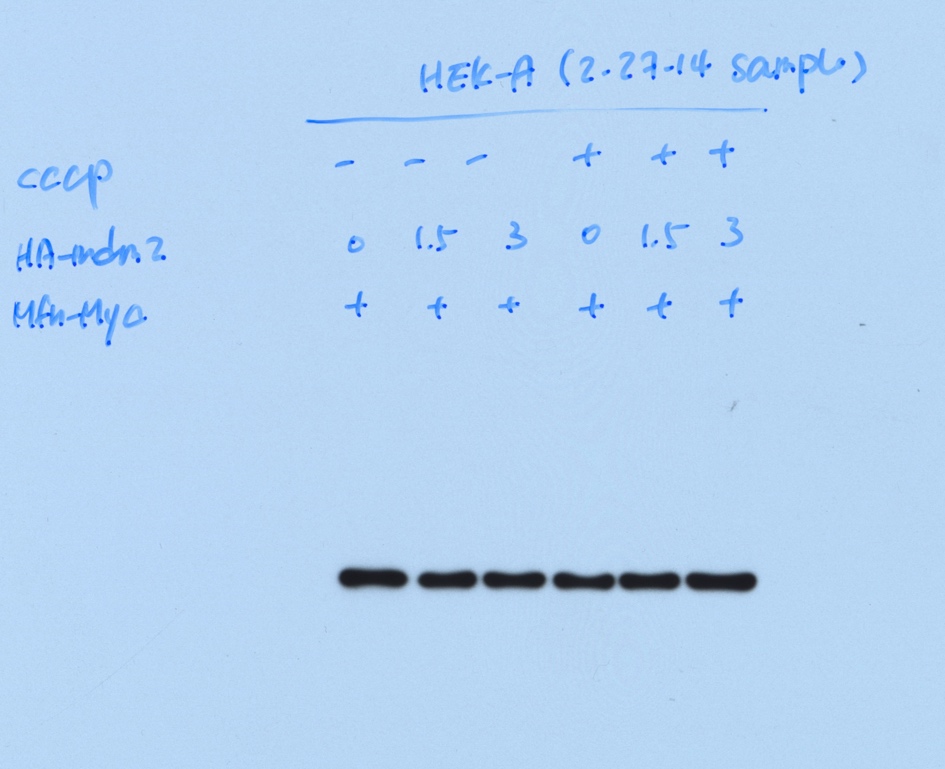


**Fig 4B:** GABDH


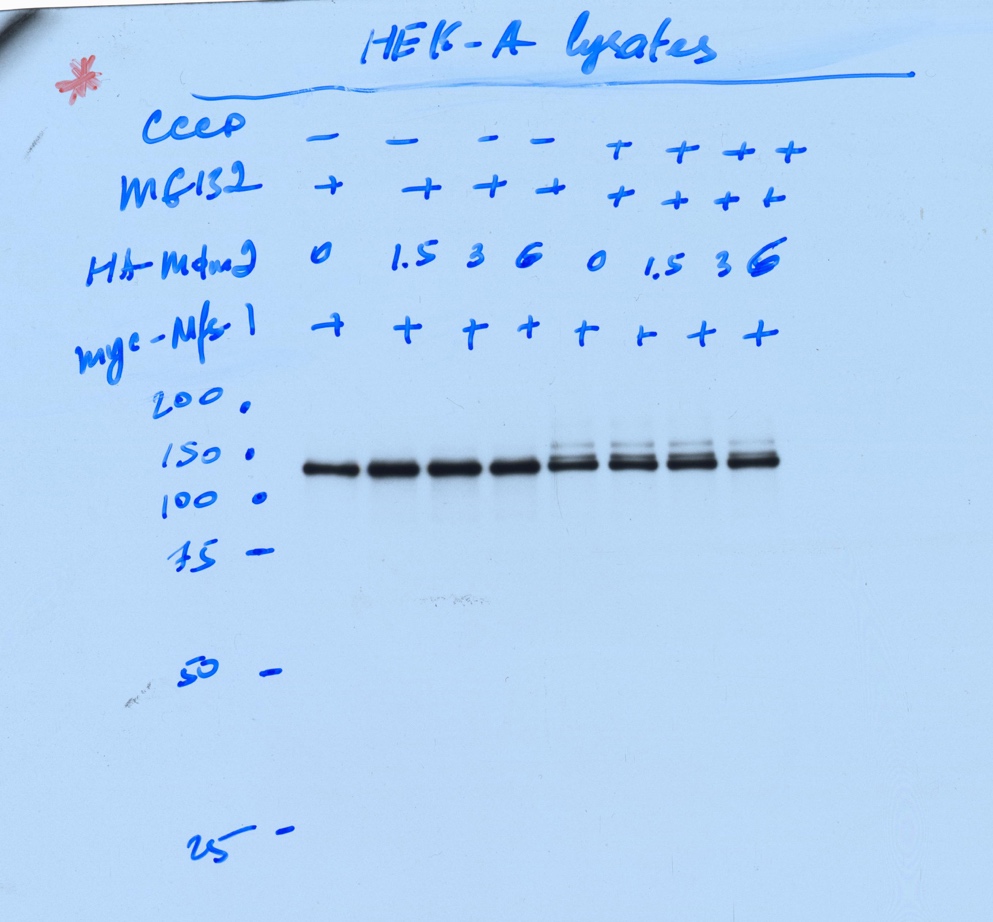


**Fig 4D upper panel:** IB myc (myc-Mfs-1)


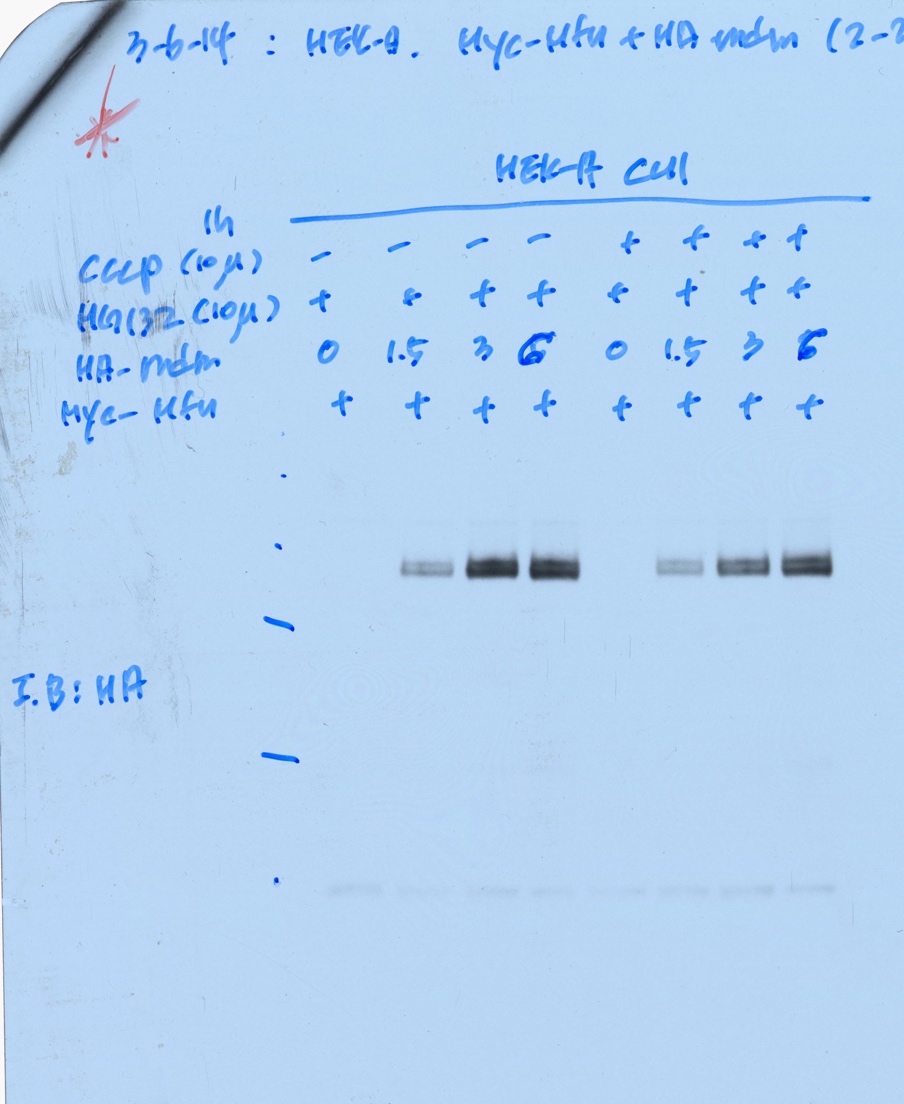


**Fig 4D upper panel:** IB HA (HA-Mdm2)


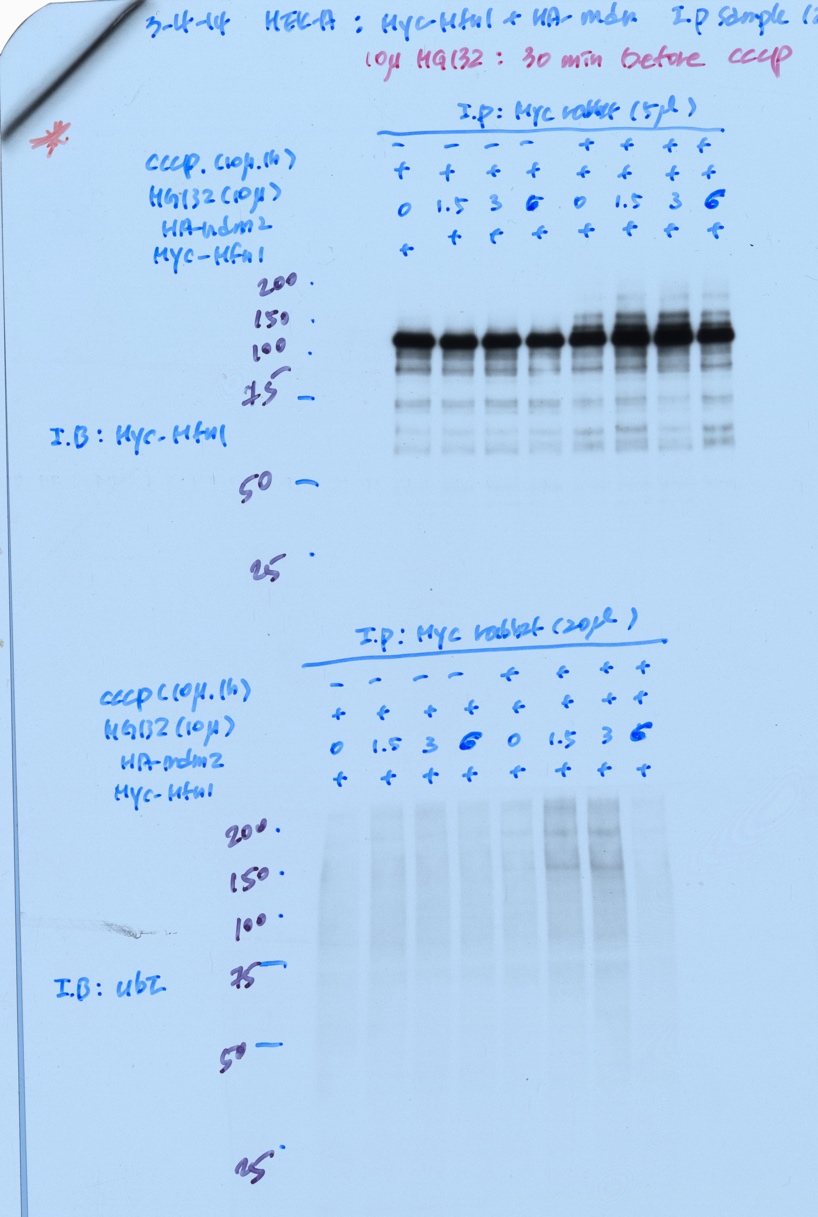


**Fig 4D lower panel:** IB myc and IB Ubiquitin

**FIGURE 6**

**
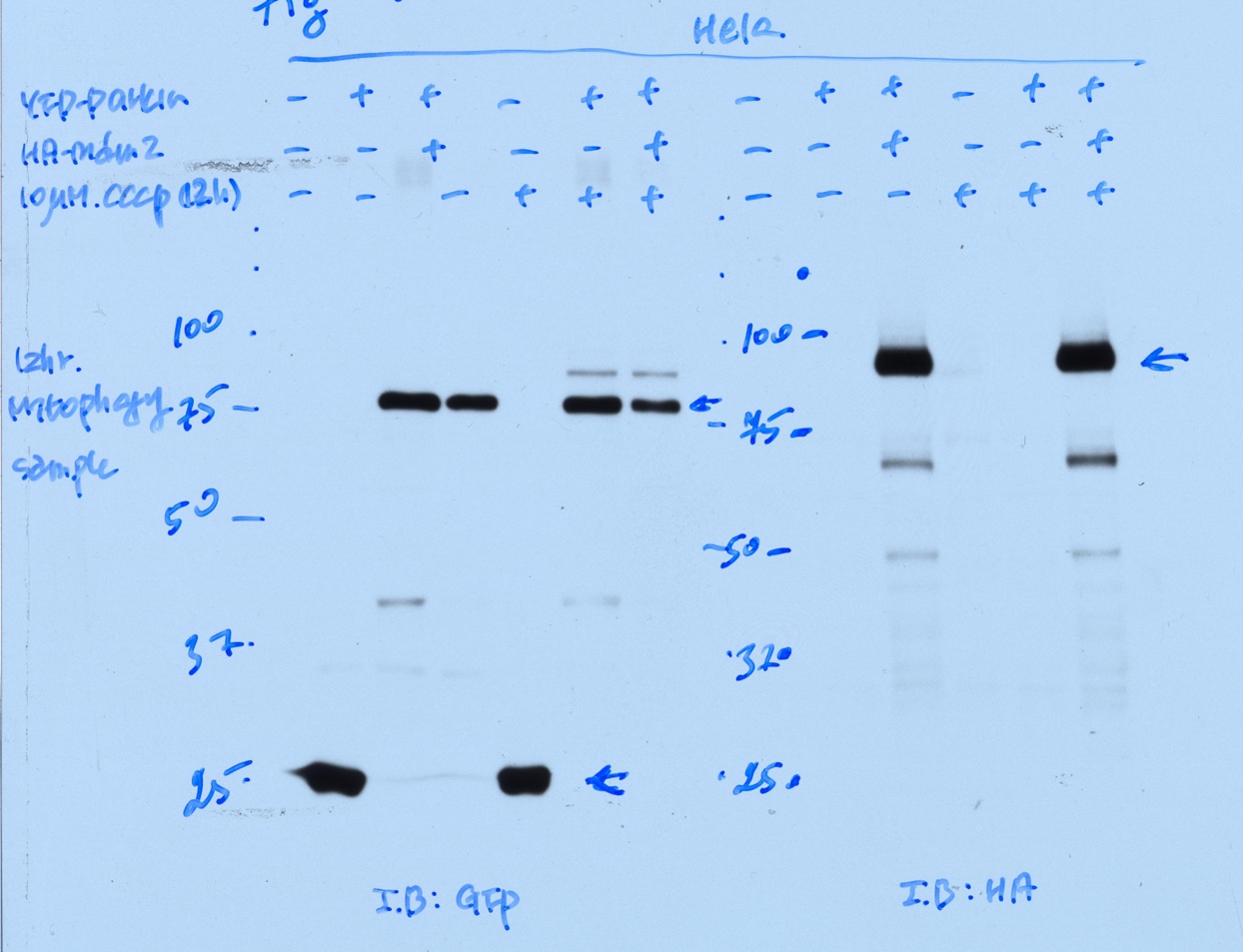
**

**Fig 6B:** Left panel **-** IB GFP (upper band - YFP-parkin; lower band - GFP). Right panel - IB HA (HA-Mdm2).


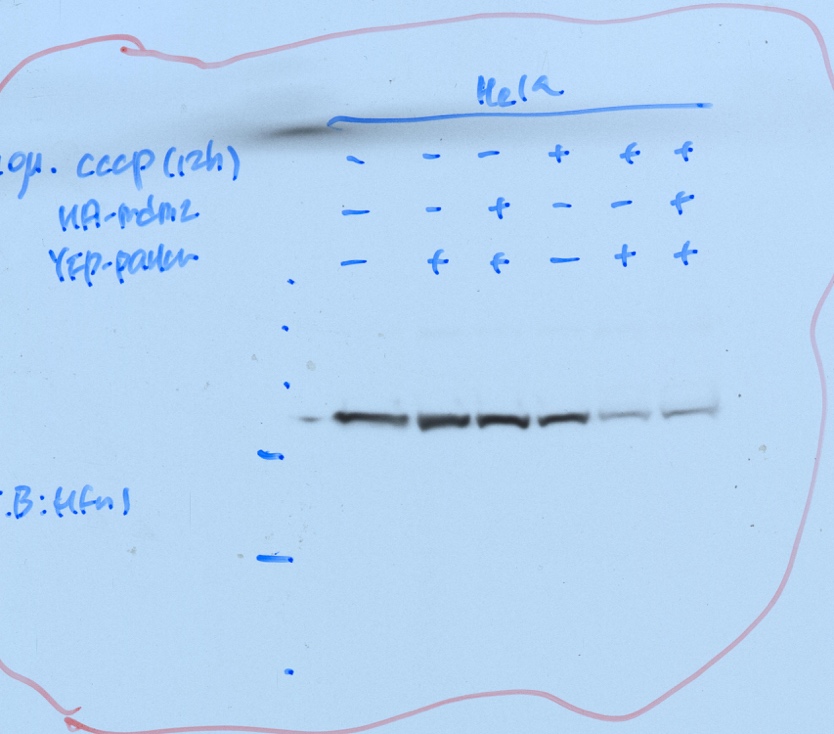


**Fig 6B:** IB Mfs-1


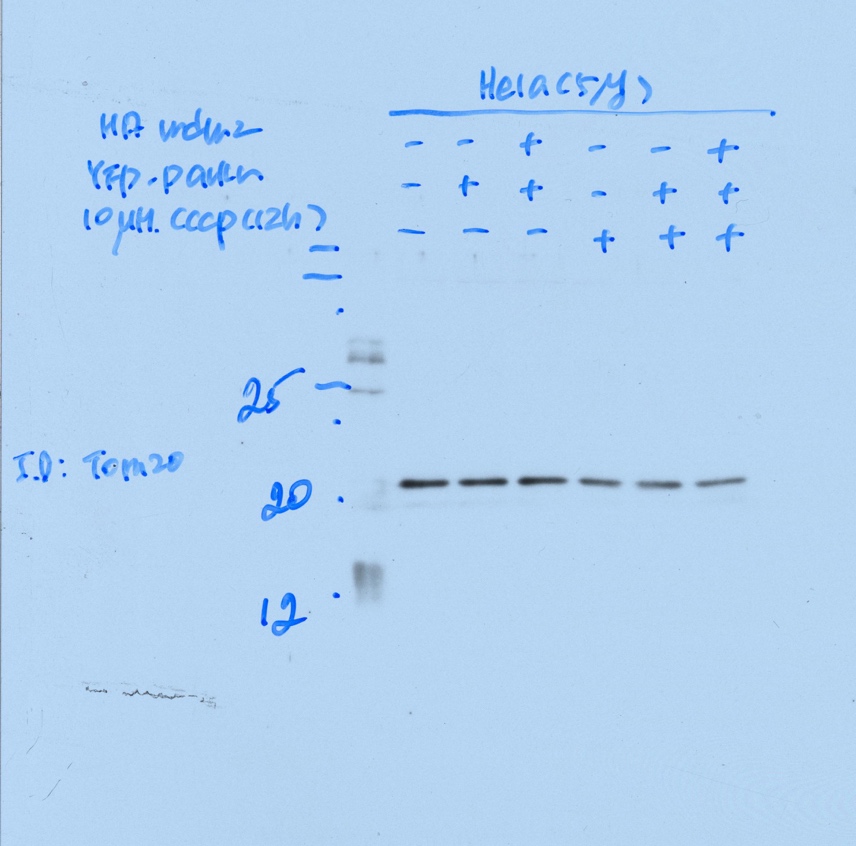


**Fig 6B:** IB Tom20.


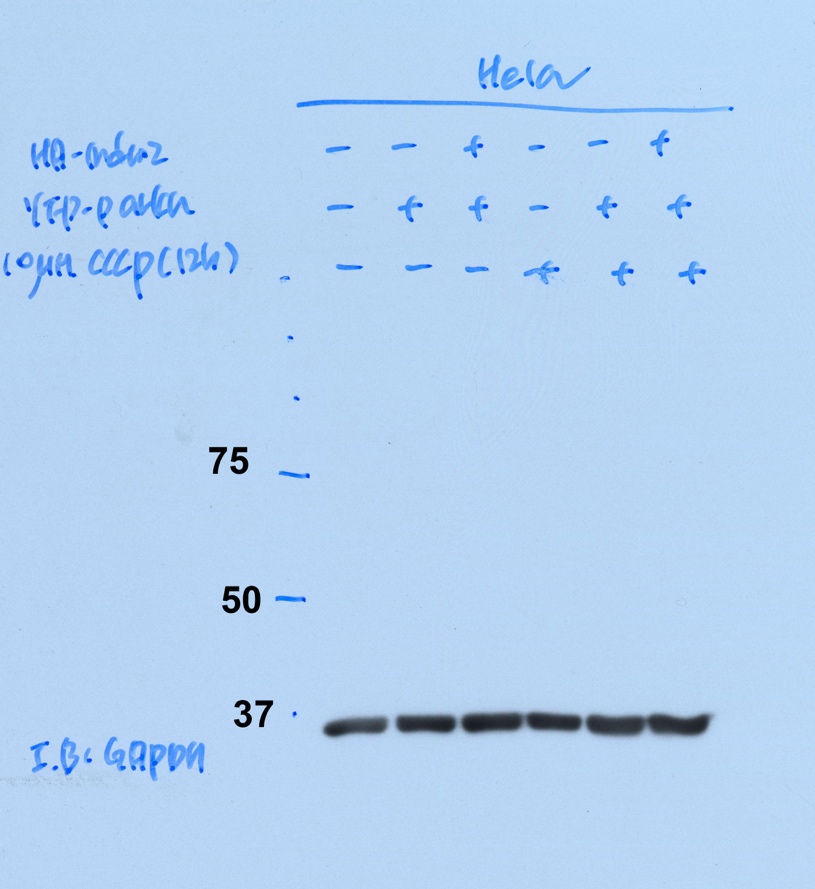


**Fig 6B:** IB GAPDH.

**FIGURE 7**


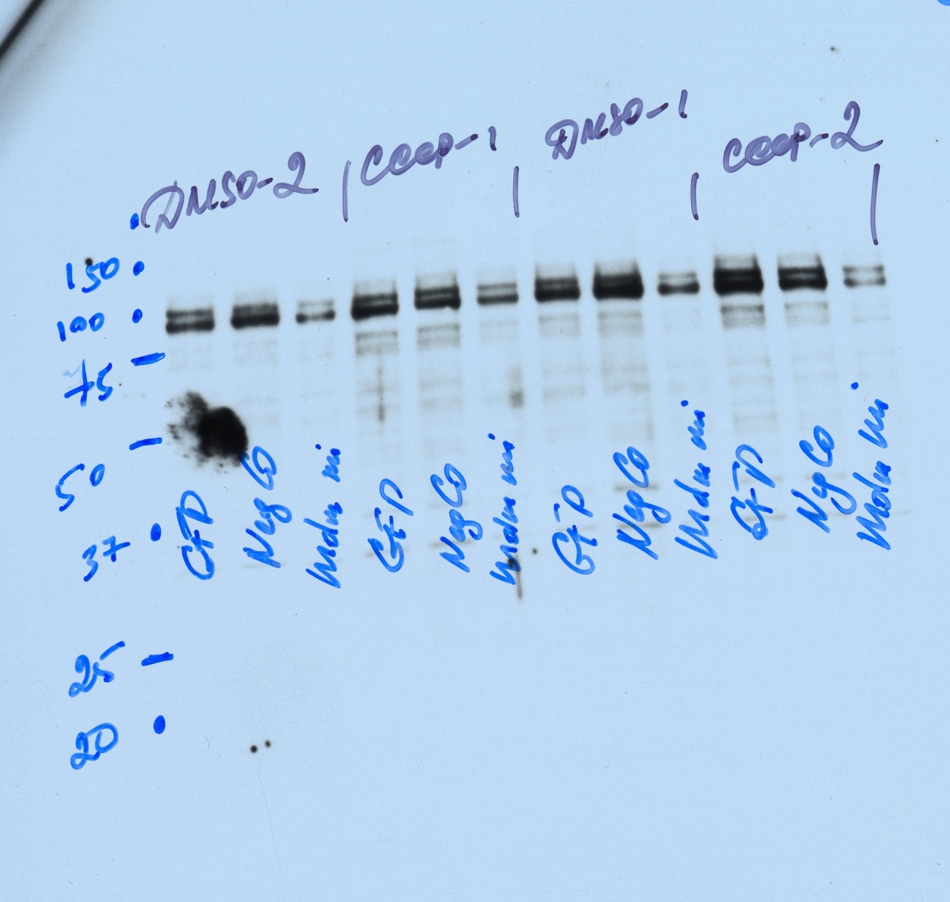


**Fig 7B:** Mdm2


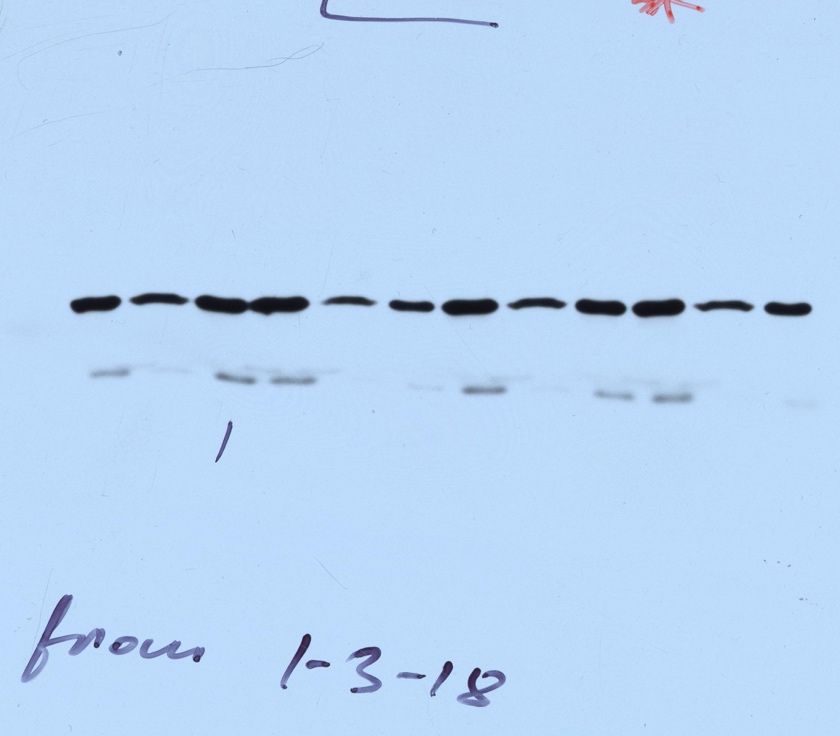


**Fig 7B:** GFP


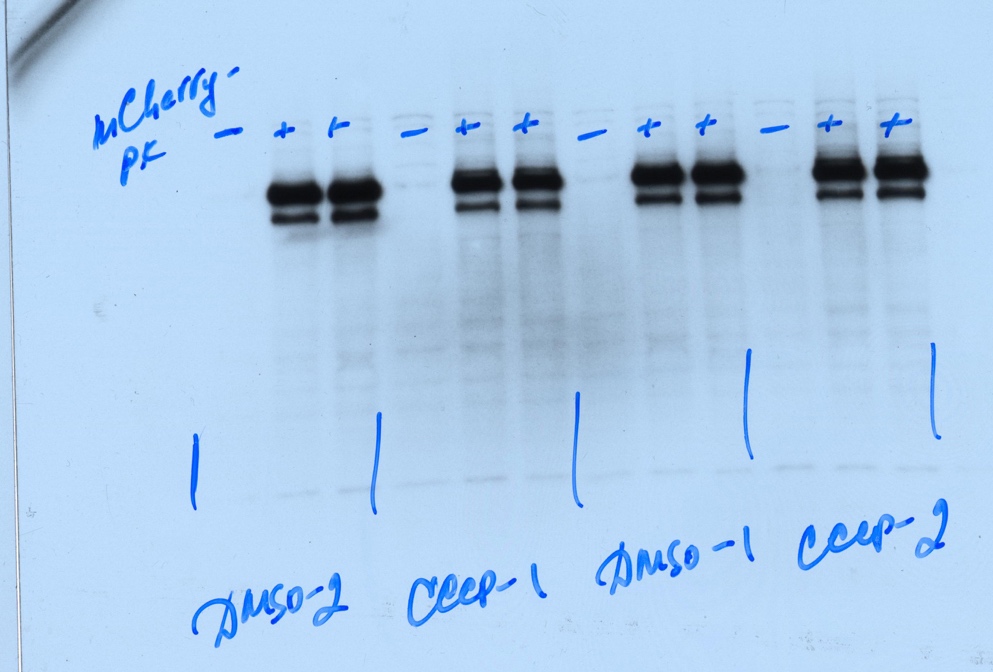


**Fig 7B:** Parkin
